# Morphometric changes on dung beetle *Dichotomius problematicus* (Coleoptera: Scarabaeidae: Scarabaeinae) related to conversion of forest into grassland: A case of study in the Ecuadorian Amazonia

**DOI:** 10.1002/ece3.9831

**Published:** 2023-02-17

**Authors:** Diego Marín‐Armijos, Adolfo Chamba‐Carrillo, Karen M. Pedersen

**Affiliations:** ^1^ Departamento de Ciencias Biológicas y Agropecuarias Colección de Invertebrados Sur del Ecuador, Museo de Zoología CISEC‐MUTPL, Universidad Técnica Particular de Loja Loja Ecuador; ^2^ Programa de Posgrado en Biodiversidad y Cambio Climático Universidad Indoamérica Quito Ecuador; ^3^ Technische Universität Darmstadt Darmstadt Germany

**Keywords:** bioindicator, climate change, functional diversity, intraspecies, land conversion, pasture, trait plasticity biodiversity, trait plasticity

## Abstract

The conversion of forest into grassland can induce differentiation in the functional morphology of resilient species. To assess this effect, we have chosen a dung beetle *Dichotomius problematicus,* as a model species. We established 20 sampling points distributed along a transect for a forest and grassland located in the Podocarpus National Park in Ecuador. Four pit‐fall traps were baited with pig feces per sample point and were left open for 48 h. We sexed and measured 13 morphological traits of 269 individuals. Nonmetric multidimensional scaling was carried out to evaluate the influence of habitat and sexual dimorphism on the traits. We applied a principal component analysis to evaluate the morphological features that best explain the differences between land use and sexual dimorphism. We used generalized linear models to evaluate the explanatory variables: habitat and sexual dimorphism with respect to morphological traits. Five traits contributed over 70% body thickness, Pronotum width, Pronotum length, Head width and Elytra length, following the results of a principal component analysis. Both habitat and sex influence traits. In the forest, the individuals are larger than grassland likely due to available resources, but in grassland, the structures in charge of the burial process head, protibia are larger, displaying a strong pronotum and possible a greater reproductive capacity given by spherecity. These patterns of changes in the size of beetles and their structures could reflect the conservation state of an ecosystem.

## Graphical abstract

We investigated the effects of land use conversion from forest into grassland on the functional morphology of dung beetles Dichotomius problematicus. This conversion can induce differentiation in the functional morphology of resilient species.

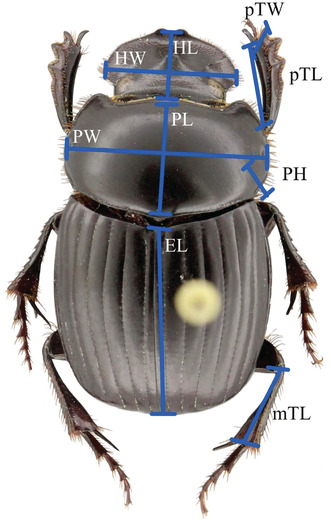

## 1 INTRODUCTION

Increased land conversion of ecosystems from natural to anthropogenic states as a significant driver of decreasing taxonomic biodiversity has been extensively studied, especially at the community level (Carrión‐Paladines et al., 2021; Edwards et al., 2014; Raine et al., 2018; Seibold et al., 2019). However, little is known about shifts in functional diversity with land conversion. There is evidence that the type of land use influences phenotypic plasticity in tropical dung beetles (Raine et al., 2018; Soto et al., 2019). These shifts could be observed in species growth rates, reproduction success, survival rates, and functional diversity (Edwards et al., 2014; Raine et al., 2018; Soto et al., 2019). Microevolutionary events influencing phenotypic traits can be observed in less than 10 generations after the triggering event (Soto et al., 2019). Land conversion, such as logging, forest fires, increased agriculture, excessive use of pesticides and herbicides, the introduction of invasive or genetically modified species, and human settlements, can create innumerable stimulus stressors, which might result in a microevolutionary event.

These microevolutionary events can be observed by changes in traits in those species resilient to environmental alterations. Traits are measurable characteristics that affect an individual's fitness, such as body size or shape. The environment might contribute to a measurable change in traits (Hernández et al., 2011). Traits can be morphological, such as body size and shape or length of body parts can be measured more simply (Soto et al., 2019). Behavioral traits can also be important. Examples include diurnal and nocturnal activity, predatory behavior (Larsen et al., 2009; Hernández et al., 2011; Martín et al., 2021), or dung processing behavior (Milotić et al., 2019). Phenological traits like the effects of seasons in the abundance or life cycles (Lobo & Cuesta, 2021; Daoudi et al., 2022; Martínez et al., 2022). Chemical traits, for instance, chemical attracts that signal predators (Goolsby et al., 2017; Martín et al., 2021). These measures and comprehension of trait diversity help us to understand and evaluate the ecosystem functioning (processes and services) and can be applied to improve decision‐making for conservation and ecosystem restoration (Cadotte et al., 2011; Gagic et al., 2015). Dung beetles are highly sensitive to environmental changes. For example, pastures present dung beetle species adapted to forest habitats with some notable environmental changes, including higher temperatures, decreased humidity, dry compact soil, types of available substance, and higher risk of predation (Barragán et al., 2014; Gómez et al., 2018). Besides, phenotypic changes by microevolution have been evaluated in *Onthophagus* species related with the emergence of horns and the increased of larval survival depending on association with microbiome, which has allowed them to adapt to different niches and environments over time (Hu et al., 2020). As well as, the changes in color polymorphism in *Onthophagus proteus* driven by environmental factors as altitude (Stanbrook et al., 2021). However, it is important to note that these are recent discoveries and need more study time (Hu et al., 2020).

Changes in body size traits may not be uniform within species in response to the same environmental changes. Males and females are sexually dimorphic, with females being larger than males (Stillwell et al., 2010). Over time, we can find evidence of the advantages of size in individual fitness, such as mating success, fecundity, or growth. For example, a large body size promotes sexual success and fecundity (Blanckenhorn, 2005). However, small male individuals also have advantages because they require less food in ecosystems with low food availability consequently, they are successful in finding a mate and reproducing (Blanckenhorn, 2005; Stillwell et al., 2010). Likewise, larger females have high fecundity producing many offspring during their life (Stillwell et al., 2010). Morphological changes include food selection which may vary depending on sex, age, or physiological condition (Salomão et al., 2022). Therefore, plasticity of sexual dimorphism concerning body size can differ between sexes and could depend on environmental variables, creating different patterns in sexual dimorphism in different population subject to different environments (Teder & Tammaru, 2005; Stillwell et al., 2010).

Environment plays a vital role in adjusting a particular insect morphological trait to occupy a niche. In arid environments, to reduce desiccation, flightless species exhibit rounder, truncated elytra in comparison with flying species (Stanbrook et al., 2021). Adults of dung beetles body size decreased with increasing temperature throughout development. This decreases energetic costs and helps beetles conserve energy for feeding, reproduction, and immune responses (Carter & Sheldon, 2020; Mamantov & Sheldon, 2021). Likewise, variation in the hind leg and eye size is consistent with temperature exposure, resource availability, and habitat structure. Large‐eyed individuals of dung beetles are abundant in logged forests that old growth forests (Raine et al., 2018). Additionally, the coloration of the body of dung beetles responds to altitudinal change. All these modifications are mechanisms for thermoregulation (Stanbrook et al., 2021). The principal traits related to dung beetles ability to remove dung are body mass and body size. The effectiveness of dung burial is influenced most by traits related to the prothorax (volume, pronotum length, and width) along with the protibial area is important (deCastro‐Arrazola et al., 2020). These traits consider the key dung exploitation strategies, especially the tibial shape, which is an evolutionary marker that changes in relation to competition or adaptation strategy (Macagno et al., 2016). Differences have also been seen in females of *Dichotomius,* where they have been found individuals with four protuberances in disturbed areas and two protuberances in forested areas (Pardo‐Diaz et al., 2019). Although, the species are known to be habitat generalists and have preferences for forest edges and grasslands as they prefer cow and horse feces (Amat‐Garcia et al., 1997; Amézquita et al., 1999; Sarmiento‐Garcés & Amat‐García, 2009b).

In this study, we investigated the effects of the forest conversion into grassland through the functional morphology of dung beetles *Dichotomius problematicus*. Habitat conversion can induce differentiation in the functional morphology of resilient species. Therefore, we have set the following aims: (1) to estimate the effect of the conversion over morphological traits and sexual dimorphism of *D. problematicus*; (2) to compare the phenotypic differences between individuals inhabiting native forest and grassland. We expect differences between individuals of both habitats according to body size and shape. In addition, we expect to find a positive relationship between the size of individuals and habitat, particularly in forest, because there is a greater availability of food resources.

## 2 METHODS

### 2.1 Study species: *Dichotomius problematicus*

The genus *Dichotomius* (Scarabaeidae: Scarabaeinae) are endemic dung beetles from the New World, consisting of approximately 170 named species (Schoolmeesters, 2021) that are distributed from the northeastern United States to central Argentina and the highest diversity is found in tropical South America (Nunes & Vaz‐de‐Mello, 2020; Rossini & Vaz‐de‐Mello, 2020). They are paracoprids and the genus is divided into four subgenera: *Dichotomius* s. str.; *Homocanthonides* Luederwaldt, 1929; *Selenocopris* Burmeister, 1849 and *Luederwaldtinia* Martínez, 1951. The genus uses both dung and carrion for alimentation. They are habitat generalists living on open areas, grassland, and forest edges with and show a preference for cattle and horses feces (Sarmiento‐Garcés & Amat‐García, 2009a; Nunes & Vaz‐de‐Mello, 2020). The length of their body usually measures between 8 and 26 mm. Due to large size and nidification habit, they efficiently remove larger amounts of dung from the soil surface than rollers and dwellers (Monteiro et al., 2020). Their eggs and food are deposited beneath the soil surface in a gallery complex, where deposition depth depends on species, type of soil, and environmental temperature (Hanski & Cambefort, 1991; Macagno et al., 2016; Monteiro et al., 2020).

*Dichotomius problematicus* (Rossini & Vaz‐de‐Mello, 2020)is in the IUCN category of Data Deficient (DD) (Vaz‐de‐Mello et al., 2014). There is a lack of information basic about the specific ecological or population dynamics of *D. problematicus*. More research is needed for taxonomic verification of the status of the species or subspecies and to make a more informed assessment based on its distribution, population dynamics, and life history patterns. Within Ecuador *Dichotomius,* species have been reported in Loja (Piscobamba), Morona Santiago, Napo, Pastaza, Sucumbíos, Tungurahua, and Zamora Chinchipe (Chamorro et al., 2019). *Dichotomius problematicus* is widely distributed in Colombia, Ecuador, and Peru.

### 2.2 Study area

The study was carried out in the Podocarpus National Park (PNP), located between the provinces of Zamora Chinchipe and Loja, south of Ecuador. The PNP has an altitudinal range from 900 to 1600 m a.s.l. and comprises an area of 146,280 hectares. The region has a humid climate >90% with mean annual precipitation ranging from around 2000 mm to 4500 mm from 1050 mm at 3060 m a.s.l. (Moser et al., 2007). Precipitation exhibits a bimodal pattern with a significant rainy season from April to July and a less intensive rainy season from September to December (Bendix et al., 2006). Annual mean air temperature decreases with increasing elevation from 19.4°C to 9.4°C (Moser et al., 2007). Podocarpus National Park is considered a megadiverse area due to its high degree of endemism and the number of species that it harbors (Thormann et al., 2018).

Sampling was conducted at the same time between November 2013 and April 2014, for which two habitats were selected: (1) Forest: located in the Bombuscaro (4°06′50″ S, 78°58′00″ W, 950 m a.s.l.), within the PNP in the province of Zamora Chinchipe, and (2) Grassland: located in Timbara (4°03′48″ S, 78°54′42″ W, 1100 m a.s.l.), within the PNP in the province of Zamora Chinchipe. It has an area of 12,750.61 hectares, of which the majority are areas of extensive livestock production. Another part is used for agricultural or subsistence production. This area has a high degree of anthropic intervention due to agricultural activities and the advance of the agricultural frontier.

### 2.3 Sampling coprophagous beetles

At each habitat site forest and intensive agriculture, we established 20 sampling points distributed along a transect and separated from each other every 50 meters (Larsen & Forsyth, 2005). At each sampling point, four pit‐fall traps were installed and distanced from each other by 1 m. The pit‐fall traps consist of a plastic container placed on the ground level and filled one‐third of the way with a mixture of water and detergent. Traps were baited with pig feces (20 g) to attract coprophagous species and were left open for 48 hours. Both habitat sites were sampled the same day. The beetles were preserved in a 90% alcohol solution and subsequently identified at the most specific taxonomic level through taxonomic keys (Chamorro et al., 2018, 2019). The collected material was deposited in the Collection of Insects of the South of Ecuador (CISEC‐MUTPL) of the Universidad Técnica Particular de Loja.

### 2.4 Morphometric measurement

**FIGURE 1:**
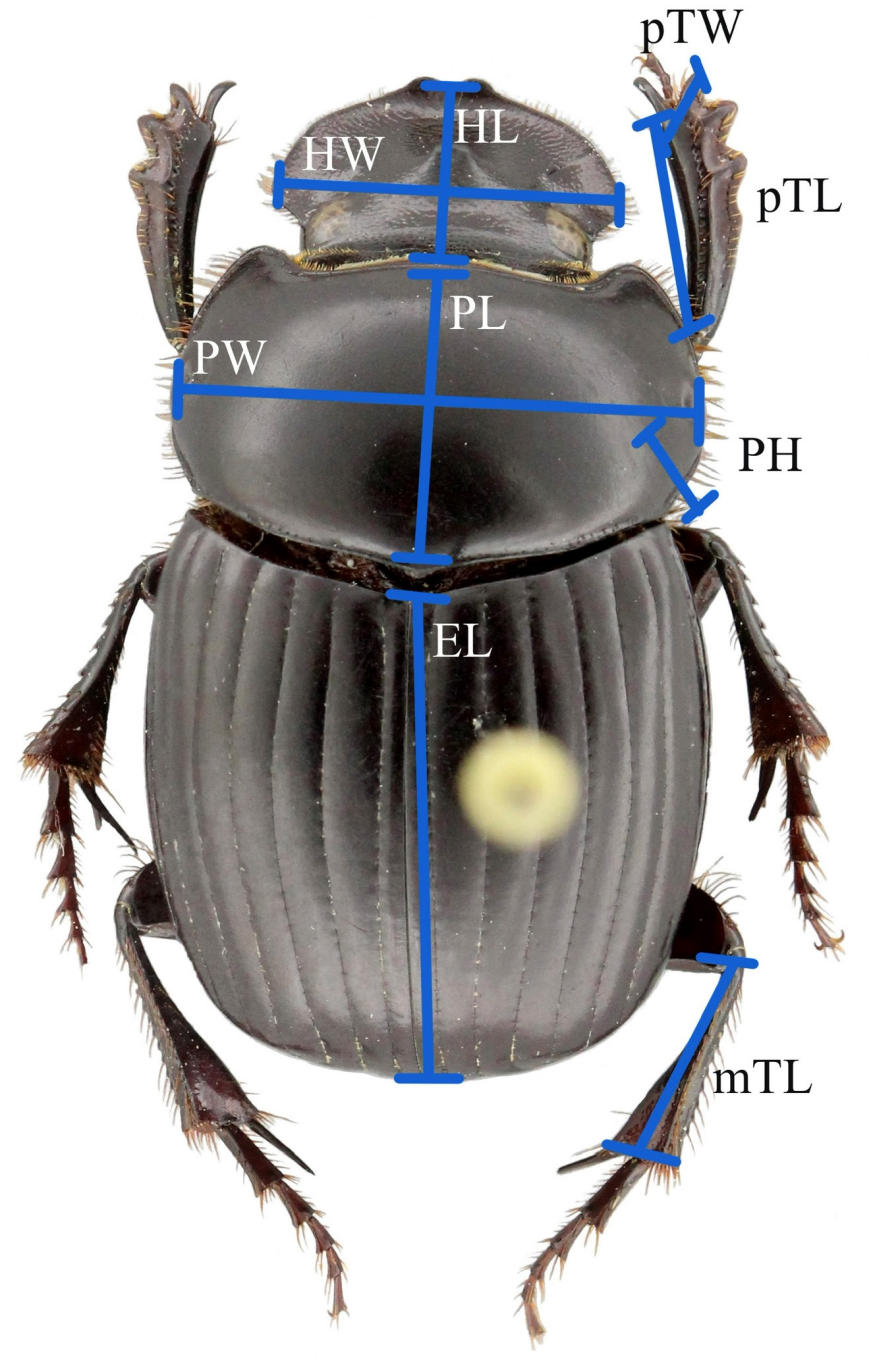
Description of the nine principal traits measured: head width (HW), head length (HL), pronotum width (PW), pronotum length (PL), pronotum high (PH), elytra length (EL), protibia width (pTW), protibia length (pTL), and metatibia length (mTL).

To estimate the functional traits of the *Dichotomius problematicus*, we measured relevant morphological characteristics of all individuals (*n* = 269, 47 from forest and 222 from grassland). The quantitative measurements were as follows (Figure 1): head width (HW), head length (HL), pronotum width (PW), pronotum length (PL), pronotum height (PH), elytra length (EL), protibia length (pTL), protibia width (pTW), metatibia length (mTL), total length (L), width elytra (I), and body thickness (S) (Soto et al., 2019). In addition, the individuals were sexed, using the shape of the tibial spur and genitalia characters depending on which trait or combination of traits was most useful. An Olympus SZ61 stereoscope with micrometric ocular measurement was used to measure the morphological features in millimeters. The sphericity was determined through the formula suggested by Sneed and Folk (Sneed & Folk, 1958) (Soto et al., 2019): $\sqrt[{3}]{\mathrm{SI}/L^{2}}$ where *L* total body length (long axis), *I* is elytra width (intermediate axis), *S* is body thickness (shaft).

### 2.5 Data analysis

All the analyses were carried out with R statistical program (R‐Core‐Team, 2019). A nonmetric multidimensional scaling (NMDS) was used as an exploratory analysis to evaluate the influence of habitat (Forest and Grassland) and sexual dimorphism (Male and Female) on the morphological traits of individuals, with the metaMDS function of the Vegan package (Oksanen et al., 2019). Before this analysis, these data were standardized with the decostand function of the Vegan package (Oksanen et al., 2019).

Then, a permutational variance analysis (PERMANOVA) was performed with the adonis function of the Vegan package (Oksanen et al., 2019) to compare the differences between groups formed in the NMDS according to habitat and sexual dimorphism.

Additionally, to visualize the results, we applied a principal component analysis (PCA) using the Bray–Curtis dissimilarity to evaluate the morphological features that best explain the differences between land use and sexual dimorphism. These data were previously standardized to guarantee uniformity. Principal component analysis was calculated with the MASS statistical package (Venables & Ripley, 2002).

Finally, the level of significance was established for each of the morphological features in relation to two explanatory variables: (1) habitat (forest and grassland) and (2) sexual dimorphism (male and female) for which a generalized linear models (GLM's) were used, through the glm function with a Gaussian distribution with the link function “identity.” Generalized linear models were calculated with the MASS statistical package (Venables & Ripley, 2002). A chi‐squared test was performed to evaluate the significance of the explanatory variables: habitat and sexual dimorphism.

## 3 RESULTS

In total, 269 individuals were measured (47 in forest and 222 in grassland) (Table S1). The average values of size in relation to habitat and sexual dimorphism are similar. However, the total length (L) presents the highest value for both forest and grassland and between females and males (Table 1; Figure 3a,b). The spherecity is higher in grassland but is not different between males and females (Table 1; Figure 4a,b).

**TABLE 1:** Mean and standard error values of the morphological traits of *Dichotomius problematicus* concerning habitat and sexual dimorphism.

Trait	Habitat			Sexual dimorphism		
	Grassland	Forest		Males	Females	
	Mean	Mean	*p* value	Mean	Mean	*p* value
Head width (HW)	3.386 ± 0.333	3.87 ± 0.547	**<.001**	3.45 ± 0.478	3.489 ± 0.359	.403
Head length (HL)	5.909 ± 0.426	5.743 ± 0.513	**.021**	5.889 ± 0.519	5.871 ± 0.365	.746
Pronotum width (PW)	5.252 ± 0.378	4.923 ± 0.337	**<.001**	5.256 ± 0.4	5.136 ± 0.375	**.008**
Pronotum length (PL)	8.88 ± 0.714	8.409 ± 0.972	**<.001**	8.879 ± 0.732	8.722 ± 0.825	.093
Pronotum high (PH)	5.17 ± 0.663	4.626 ± 0.736	**<.001**	5.147 ± 0.753	5.006 ± 0.654	.087
Elytra length (EL)	7.986 ± 0.604	8.255 ± 0.672	**.009**	7.995 ± 0.64	8.07 ± 0.607	.315
Elytra width (I)	9.412 ± 0.653	9.359 ± 0.739	.565	9.360 ± 0.699	9.444 ± 0.637	.305
Protibia width (pTW)	1.395 ± 0.229	1.34 ± 0.269	.099	1.351 ± 0.265	1.419 ± 0.202	**.018**
Protibia length (pTL)	3.768 ± 0.402	3.526 ± 0.493	**<.001**	3.751 ± 0.49	3.701 ± 0.36	.332
Metatibia length (mTL)	4.03 ± 0.294	4.06 ± 0.468	.708	3.985 ± 0.343	4.083 ± 0.311	**.016**
Total length (L)	16.624 ± 0.893	17.049 ± 1.146	**.005**	16.702 ± 0.988	16.696 ± 0.923	.959
Body thickness (S)	7.173 ± 0.588	6.401 ± 0.657	**<.001**	6.976 ± 0.67	7.096 ± 0.662	.101
Sphericity (Sph)	0.11 ± 0.008	0.086 ± 0.015	**<.001**	0.104 ± 0.013	0.107 ± 0.013	**.029**

Statistically significant values are indicated in bold (*p* < .01).

**FIGURE 2:**
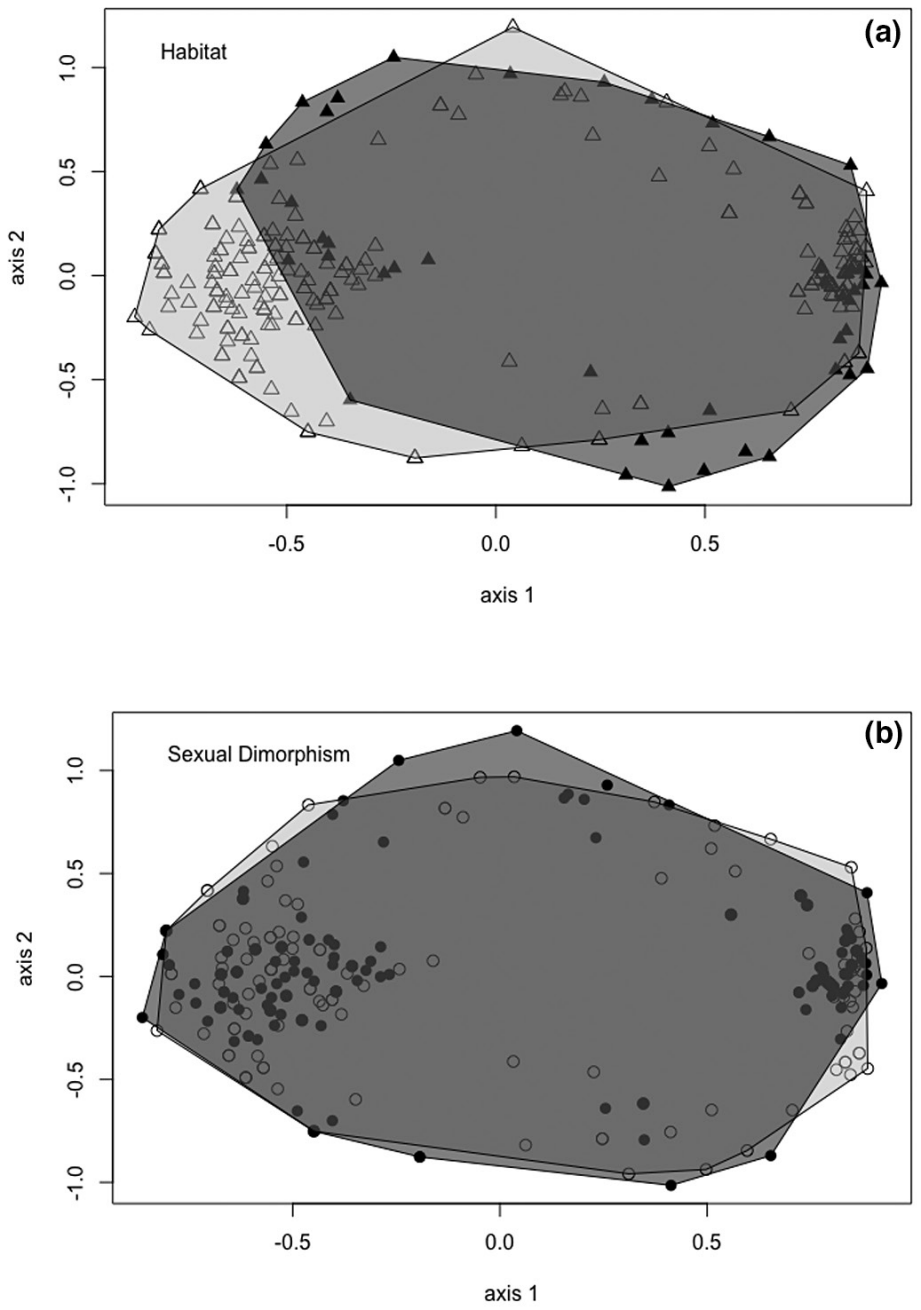
Nonmetric multidimensional scaling (NMDS) based on nine morphological traits of *Dichotomius problematicus* according to habitat and sexual dimorphism in a humid tropical forest in southern Ecuador. (a) The polygon with black triangles represents individuals from the forest and the polygon with empty triangles represents individuals from the grassland. (b) The polygon with the black circles represents males and the empty circles represent females.

Regarding the type of habitat, the NMDS (stress = 0.2) and the PERMANOVA separated the individuals (*F* = 16.53, *r* ^2^ = .055, *p* < .001) (Figure 2). Sexual dimorphism was not observed (*F* = 0.523, *r* ^2^ = .002, *p* = .690) nor is there any apparent interaction between habitat and sex (*F* = 0.5797, *r* ^2^ = .002, *p* = .583). The ordering of the NMDS shows that the morphological traits measured are partially overlapping with a significant subset of the beetles from grasslands differing from beetles in forest habitats (Figure 2a). However, when comparing traits for *D. problematicus* with NMDS, traits are completely overlapping between male and female beetles (Figure 2b). These patterns show that there are greater morphological differences (10 of 13 variables) associated with habitat than with sexual dimorphism (four of 13 variables) (Table 1).

**TABLE 2:** Mean and standard error values of the morphological traits of *Dichotomius problematicus* concerning sexual dimorphism in function of habitat.

Trait	Grassland			Forest		
	Male	Female		Male	Female	
	Mean	Mean	*p* value	Mean	Mean	*p* value
Head width (HW)	5.889 ± 0.482	5.928 ± 0.361	.498	5.884 ± 0.715	5.646 ± 0.289	.120
Head length (HL)	3.411 ± 0.510	3.370 ± 0.192	.434	3.742 ± 0.638	3.957 ± 0.469	.189
Pronotum width (PW)	8.915 ± 0.713	8.845 ± 0.716	.468	8.663 ± 0.825	8.237 ± 1.038	.140
Pronotum length (PL)	5.304 ± 0.378	5.199 ± 0.372	**.037**	4.974 ± 0.420	4.889 ± 0.270	.405
Pronotum high (PH)	5.232 ± 0.716	5.106 ± 0.602	.158	4.647 ± 0.792	4.611 ± 0.709	.869
Elytra length (EL)	7.951 ± 0.621	8.023 ± 0.585	.377	8.253 ± 0.700	8.257 ± 0.665	.982
Elytra width (I)	9.401 ± 0.989	9.484 ± 0.620	.468	9.471 ± 0.820	9.283 ± 0.683	.389
Protibia width (pTW)	1.362 ± 0.271	1.430 ± 0.170	**.026**	1.289 ± 0.223	1.375 ± 0.295	.290
Protibia length (pTL)	3.776 ± 0.490	3.760 ± 0.286	.769	3.605 ± 0.472	3.471 ± 0.508	.367
Metatibia length (mTL)	3.977 ± 0.343	4.084 ± 0.221	**.006**	4.037 ± 0.347	4.075 ± 0.541	.787
Total length (L)	16.532 ± 1.850	16.592 ± 0.845	.758	16.968 ± 1.228	17.104 ± 1.106	.696
Body thickness (S)	7.138 ± 0.657	7.234 ± 0.581	.245	6.179 ± 0.544	6.552 ± 0.692	**.054**
Sphericity (Sph)	0.108 ± 0.011	0.111 ± 0.008	**.010**	0.080 ± 0.012	0.091 ± 0.015	**.013**

Statistically significant values are indicated in bold (*p* < .01).

The results of the PCAof the 13 morphological variables we impute five were responsible for the largest degree of variation in the data (PC1 and PC2). Those five variables were consistent with contribution of variables we selected body thickness (S), pronotum width (PW), pronotum length (PL), head width (HW), and elytra width (I) that explain the 72.3% of variation (Figure 7a,b).

In the analysis of morphological traits with a GLMs, 10 significant morphological differences head width (HW), head length (HL), pronotum width (PW), pronotum length (PL), pronotum high (PH), elytra length (EL), body thickness (S), protibia length (pTL), total length (L), and sphericity (Sph) were observed for the habitat of the 13 traits measured (Table 3). With respect to sexual dimorphism, five significant morphological traits pronotum length (PL), body thickness (S), protibia width (pTW), metatibia length (mTL), and sphericity (Sph) were observed (Table 3).

**TABLE 3:** GLM analysis and Chi test of traits according to habitat (grassland and forest) and sexual dimorphism (males and females) of traits of *Dichotomius problematicus*.

Response variables	Explanatory variables	Estimator	Standard error	*t*‐value	*p*‐value
Head width (HW)	Habitat + Sexual dimorphism:				
	(Intercept)	5.905	0.040	146.146	**<.001**
	Grassland	−0.165	0.071	−2.315	**.021**
	Male	0.008	0.054	0.147	.883
	Chi test:				
	Habitat				**.020**
	Sexual dimorphism				.883
Head length (HL)	Habitat + Sexual dimorphism:				
	(Intercept)	3.392	0.038	88.678	**<.001**
	Grassland	0.479	0.068	7.097	**<.001**
	Male	−0.003	0.051	−0.051	.959
	Chi test:				
	Habitat				**<.001**
	Sexual dimorphism				.959
Pronotum width (PW)	Habitat + Sexual dimorphism:				
	(Intercept)	8.815	0.070	126.699	**<.001**
	Grassland	−0.459	0.123	−3.736	**<.001**
	Male	0.130	0.093	1.396	.164
	Chi test:				
	Habitat				**<.001**
	Sexual dimorphism				.163
Pronotum length (PL)	Habitat + Sexual dimorphism:				
	(Intercept)	5.201	0.034	154.863	**<.001**
	Grassland	−0.319	0.059	−5.372	**<.001**
	Male	0.102	0.045	2.260	**.025**
	Chi test:				
	Habitat				**<.001**
	Sexual dimorphism				**.024**
Pronotum high (PH)	Habitat + Sexual dimorphism:				
	(Intercept)	5.114	0.062	83.049	**<.001**
	Grassland	−0.533	0.109	−4.903	**<.001**
	Male	0.111	0.083	1.340	.182
	Chi test:				
	Habitat				**<.001**
	Sexual dimorphism				.180
Elytra width (I)	Habitat + Sexual dimorphism:				
	(Intercept)	9.462	0.074	127.244	**<.001**
	Grassland	−0.089	0.131	−0.674	.501
	Male	−0.035	0.100	−0.349	.727
	Chi test:				
	Habitat				.516
	Sexual dimorphism				.727
Elytra length (EL)	Habitat + Sexual dimorphism:				
	(Intercept)	8.017	0.056	142.680	**<.001**
	Grassland	0.263	0.099	2.648	**.009**

	Male	−0.060	0.075	−0.802	.424
	Chi test:				
	Habitat				**.007**
Sexual dimorphism				.423
Body thickness (S)	Habitat + Sexual dimorphism:				
	(Intercept)	7.258	0.057	127.617	**<.001**
	Grassland	−0.798	0.100	−7.944	**<.001**
	Male	−0.144	0.076	−1.888	.060
	Chi test:				
	Habitat				**<.001**
	Sexual dimorphism				**.059**
Protibia width (pTW)	Habitat + Sexual dimorphism:				
	(Intercept)	1.431	0.021	67.128	**<.001**
	Grassland	−0.062	0.038	−1.652	.100
	Male	−0.071	0.029	−2.492	**.013**
	Chi test:				
	Habitat				.143
	Sexual dimorphism				**.013**
Protibia length (pTL)	Habitat + Sexual dimorphism:				
	(Intercept)	3.750	0.038	98.120	**<.001**
	Grassland	−0.239	0.067	−3.540	**<.001**
	Male	0.036	0.051	0.700	.485
	Chi test:				
	Habitat				**<.001**
	Sexual dimorphism				.484
Metatibia length (mTL)	Habitat + Sexual dimorphism:				
	(Intercept)	4.079	0.030	136.594	**<.001**
	Grassland	0.020	0.053	0.375	.708
	Male	−0.096	0.040	−2.395	**.017**
	Chi test:				
	Habitat				.576
	Sexual dimorphism				**.017**
Total length (L)	Habitat + Sexual dimorphism:				
	(Intercept)	16.598	0.127	130.454	**<.001**
	Grassland	0.480	0.225	2.136	**.034**
	Male	−0.072	0.171	−0.424	.672
	Chi test:				
	Habitat				**.030**
	Sexual dimorphism				.671
Sphericity (Sph)	Habitat + Sexual dimorphism:				
	(Intercept)	0.112	0.001	114.344	**<.001**
	Grassland	−0.023	0.002	−13.404	**<.001**
	Male	−0.005	0.001	−3.559	**<.001**
	Chi test:				
	Habitat				**<.001**
	Sexual dimorphism				**<.001**

Statistically significant values are indicated in bold (*p* < .01).

**FIGURE 3:**
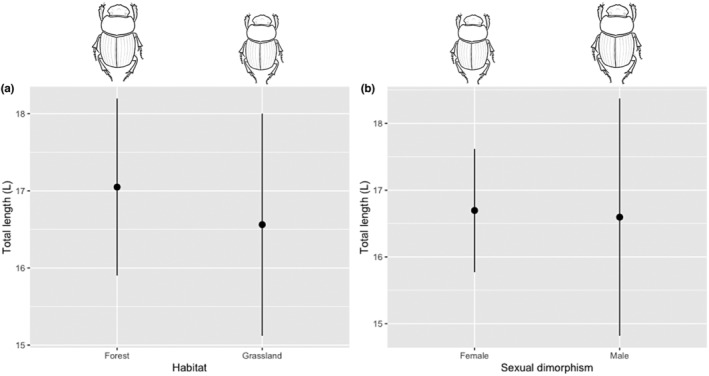
Effect of habitat (forest and grassland) (a) and sexual dimorphism (female and male) (b) the total length of *Dichotomius problematicus*, in a Tropical Forest in Ecuador (mean ± 95% confidence intervals).

**FIGURE 4:**
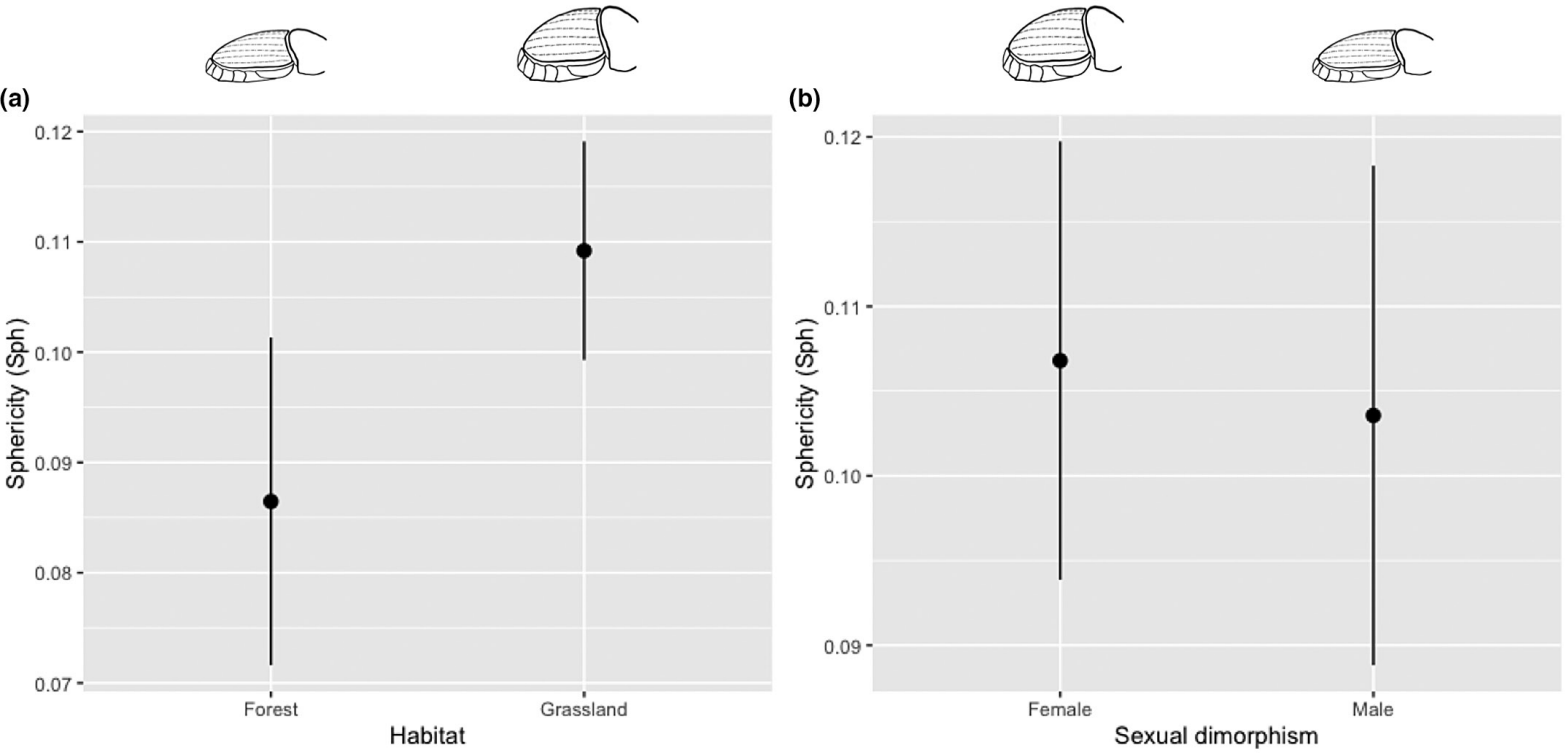
Effect of habitat (forest and grassland) (a) and sexual dimorphism (female and male) (b) on sphericity of *Dichotomius problematicus,* in a Tropical Forest in Ecuador (mean ± 95% confidence intervals).

**FIGURE 5:**
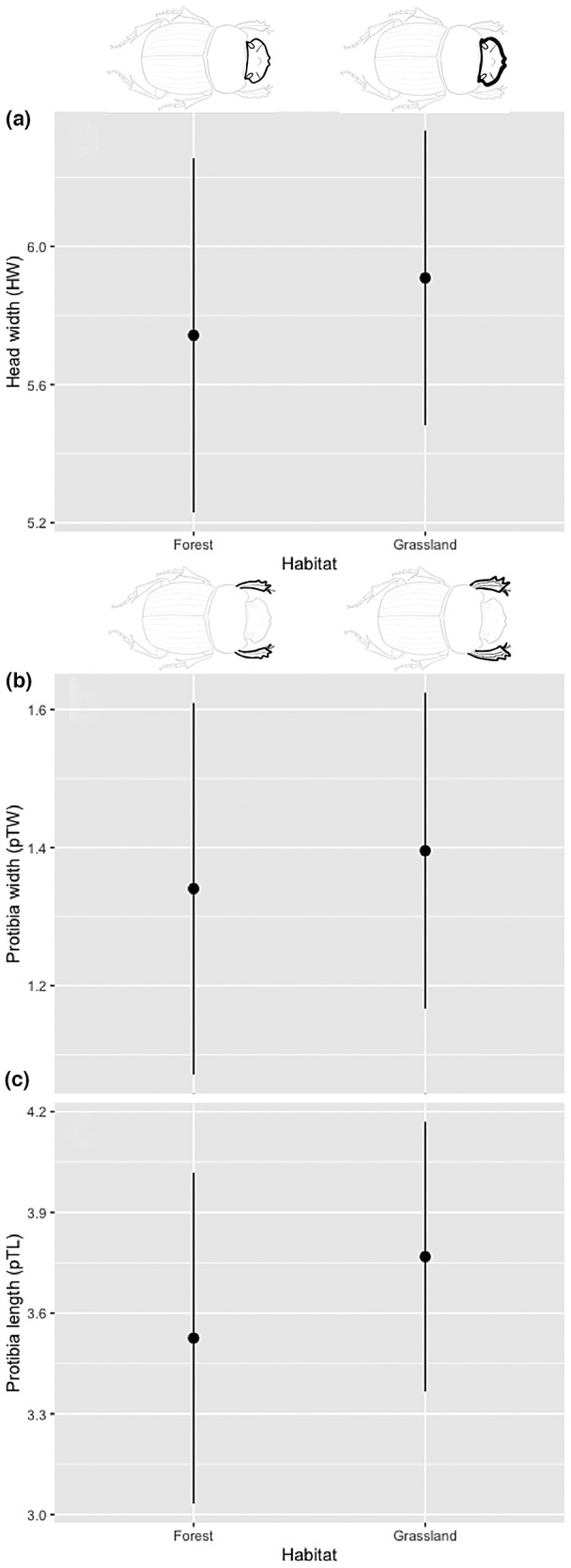
Effect of habitat (forest and grassland) on head width (a), protibia width (b) and protibia length (c) of *Dichotomius problematicus*, in a Tropical Forest in Ecuador (mean ± 95% confidence intervals).

**FIGURE 6:**
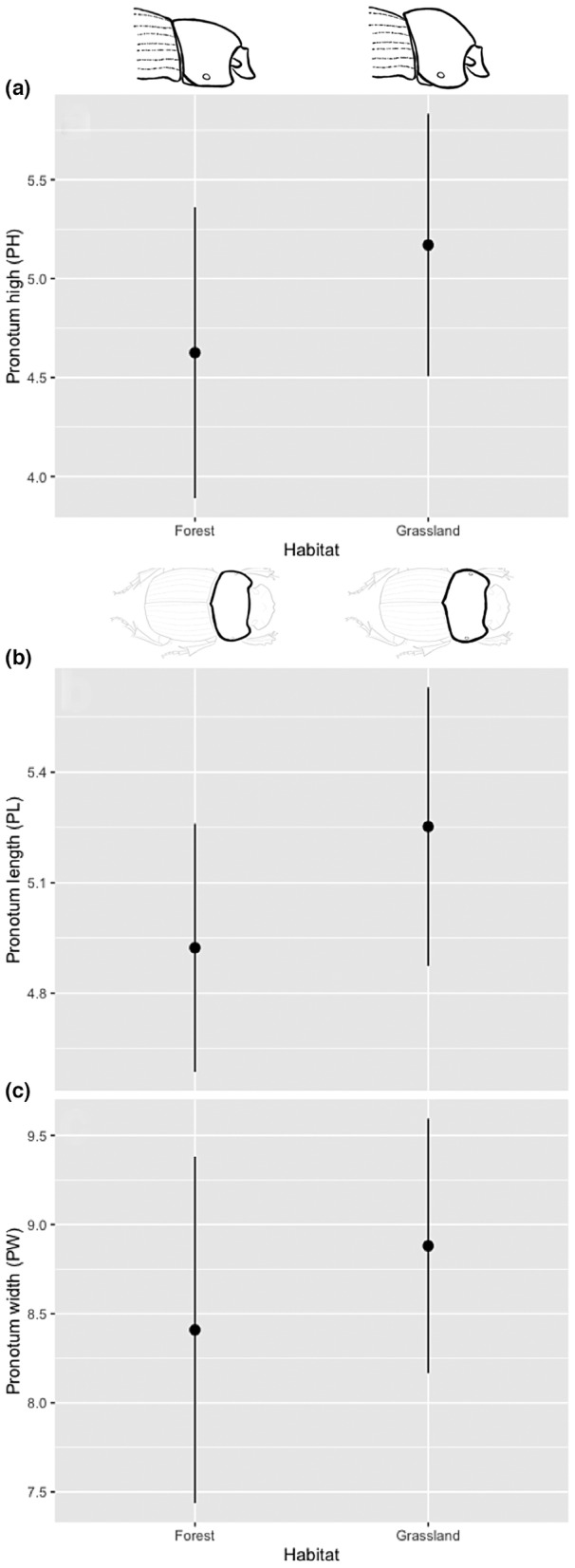
Effect of habitat (forest and grassland) on pronotum high (a), pronotum length (b) and pronotum width (c) of *Dichotomius problematicus*, in a Tropical Forest in Ecuador (mean ± 95% confidence intervals).

These results show a considerable effect on morphological traits in the conversion from forest into grassland on the individuals of *D. problematicus* (Tables 1 and 2). The individuals from the forest are longer (Figure 3a) and less spherical than those of grassland (Table 1; Figure 4a). However, the individuals from grassland tend to be smaller (Figure 3a) but with protibia length and width larger (Figure 5c,b) as well as head longer (Figure 5a) and pronotum robust than forest individuals (Figure 6a–c). With respect to dimorphism sexual, the females tend have longer metatibia than males (Table 1). In relation with the difference between males and females under the grassland—forest factor conditions, we found greater variation in traits associated with grassland: pronotum length (PL), protibia width (pTW), metatibia length (mTL), and sphericity (Sph) with respect to forest with only two traits: body thickness (S) and sphericity (Sph) (Table 2). Despite some previous publications suggesting that males particularly paracorprids are larger, we did not find this to be true in our study area (deCastro‐Arrazola et al., 2020).

**FIGURE 7:**
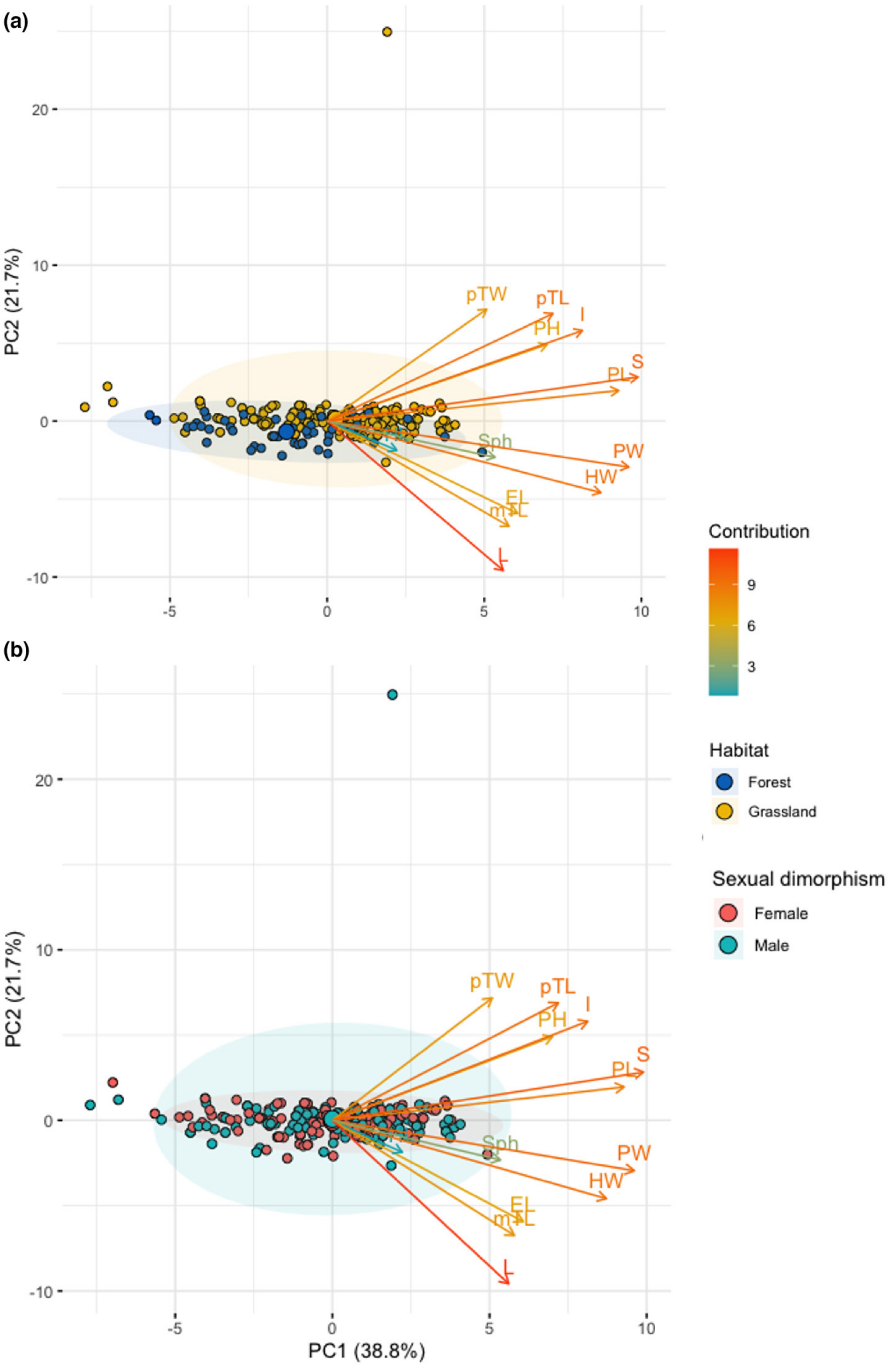
Principal component analysis of 13 morphological traits of *Dichotomius problematicus*: head width (HW), head length (HL), pronotum width (PW), pronotum length (PL), pronotum high (PH), elytra length (EL), protibia width (pTW), protibia length (pTL), metatibia length (mTL), elytra width (I), body thickness (S), total length (L) and sphericity (Sph) according to: (a) habitat (forest and grassland), and (b) sexual dimorphism (female and male).

## 4 DISCUSSION

In our study, most of the outstanding variation in morphological traits came from beetles collected in different habitats (10 of 13 traits). The results of a PCA indicate five variables with the greatest contribution: body thickness (S), pronotum width (PW), pronotum length (PL), head width (HW), and elytra width (I). Fewer traits differ between males and females only four of 13 morphological traits show significant differentiation. Of those, only two of them exhibit greater contribution: pronotum length (PL) and body thickness (S). Indicating that sexual dimorphism is a less important factor in trait differentiation than habitat in our study site. Conversion of forest to pastures has exhibited a much larger pressure on the morphological traits of *D. problematicus*. This is similar to studies of *Canthon quinquemaculatus* (in Brazil) (Alves & Hernández, 2017; Soto et al., 2019; Alves et al., 2020), and another study in Borneo (Raine et al., 2018).

Total length (L) represents a proxy for size and indicates that individuals in the forest are larger than individuals in the grasslands. This could be a result of the quantity and quality of food resources, available in pastures when compared with forests like the pattern observed in *C. quinquemaculatus* (Soto et al., 2019). Grasslands offer an abundance of cow dung that is exposed to more extreme temperature and humidity changes than in the forest. In contrast, the forests will be relatively poor in dung recourses because there are no mammals remotely as large or abundant as cows and thus offer less dung but a greater diversity of resources, possibly of higher quality. This might result in better quality nutrients for developing beetles and more choice for high‐quality nutrients for parents. Thus, the larger‐sized dung beetles are more sensitive to ecological disturbance, giving the opportunity to smaller individuals in open areas (Shahabuddin et al., 2010) or conversely there are fewer resources available to feed offspring, and they are unable to reach the size of beetles in the forest. The use of pit‐fall traps does not allow us to characterize any changes in behavior including possible increased competition for resources due to increased *D. problematicus* individuals in the pasture. We do acknowledge that there is clearly a greater density of *D. problematicus* in the grassland. However, it should not be discounted without further investigation that beetles of smaller size might result from the increased competition.

As well as, in grassland, we found individuals had longer measured, (1) head width (HW), (2) protibia width (PW), and (3) protibia length (PL) than forest individuals possibly dues to the need to be stronger for digging the hard, dry soil in open areas created by grassland. This is an important characteristic of *Dichotimius* species because they need strong and well‐developed anterior legs to build the galleries close to food resources (Hanski & Cambefort, 1991). Additionally, it is worth mentioning that the guild of paracoprids plays an important role in the removal rates of dung in grasslands (Ortega‐Martínez et al., 2021), even greater than rollers and dwellers (Monteiro et al., 2020). These results also correspond to the largest number of individuals found in grasslands, since it is known that this species has preferences for open areas, forest edges, and grasslands (Sarmiento‐Garcés & Amat‐García, 2009a; Nunes & Vaz‐de‐Mello, 2020).

Spherecity (Sph) could be associated with reproductive capacity (Raine et al., 2018) and is relatively higher in females in our study. Sphericity is also associated with tolerance to heat in open areas being less spherical than forest (Soto et al., 2019). Elytra length could be related to flying efficiency, dispersal distance, and to being higher in forest. It would be shorter in males because they might not experience the same pressure to fly longer distances to provide for their offspring (Raine et al., 2018).

The pronotum is larger in individuals of grassland that forest possibly because this part of the body are inserted legs and wings and investing more in pronotal size could increase dispersal capacity (Nunes et al., 2021). The food in nature is a limit resources (Hanski & Cambefort, 1991; Verdú & Galante, 2002), due to its patchy distribution, but high more importantly quality food resources are more important than just food resources. Exposure to wind, sun, and other competitors can reduce resource quality and availability (Hanski & Cambefort, 1991; Menéndez et al., 2014; Salomão et al., 2022). Therefore, dung beetles must arrive quickly and, in some cases, relocate the food sources to ensure their survival and that of their offspring (Hanski & Cambefort, 1991; Salomão et al., 2022).

All these changes in the species assemblage, structure, and morphology drive changes in body size, abdomen size, wing loading, hind leg size, and eye size (Raine et al., 2018), which change a species' ecological functions and their efficacy with regard to ecosystem services (functional traits) such as the quantity of manure removal (Slade et al., 2007), seed dispersal ability (Nichols et al., 2008), and biological control of parasites (Nichols et al., 2008). If a functional group (dwellers, tunnelers, or rollers) is absent greater, it is expected to exacerbate a loss or degradation of ecosystem services, which could affect the rest of the ecological network (Gardner et al., 2008; Larsen et al., 2005; Slade et al., 2007).

With all this evidence, we suggest that functional diversity based on traits is an important tool that complements the taxonomical diversity because traits reflect the state of an ecosystem at a particular time and individuals' response to the current state. These responses by the dung beetle community have downstream effects on ecosystem services such as how efficiently dung beetles will be as seed dispersers and the speed and quantity of dung removal (Gagic et al., 2015; Milotić et al., 2019; Slade et al., 2007). Previous publications (Raine et al., 2018; Soto et al., 2019) provide evidence that body size could decrease as a response to global warming because an increase in mean minimum temperature could affect the structure small individuals who will be more sensitive to these environmental changes and migrate or disappear (Milotić et al., 2019). This trait of dung beetles improves their usefulness as indicators of anthropogenic habitat disturbance, on global and local levels (Gagic et al., 2015; Hernández et al., 2011; Milotić et al., 2019).

Over this study, we found patterns of changes in the size of beetles and the structural morphology of their anterior legs, head, and pronotum changed with respect to habitat and, to a lesser degree sexual dimorphism. Thus, individuals of *D. problematicus* reflect the conservation state of an ecosystem in the south tropical forest of Ecuador where our study was conducted. Future studies should focus on investigating the microevolutionary adaptation of dung beetles addressing different habitats to improve understanding of these processes of adaptation and assess whether these changes respond in a different way to other stressors, and deepen the knowledge of their biology and ecology.

## AUTHOR CONTRIBUTIONS

**Diego Marín‐Armijos:** Conceptualization (equal). **Adolfo Chamba:** Conceptualization (equal). **Karen Pedersen:** Conceptualization (equal).

## FUNDING INFORMATION

This research received no external funding.

## CONFLICT OF INTEREST

The authors declare no conflict of interest.

## PERMISSION TO REPRODUCE MATERIALS FROM OTHER SOURCES

None.

## Supporting information


Table S1.

## Data Availability

The data that supports the findings of this study are available in the supplementary material of this article.

## References

[ece39831-bib-0001] Alves, V. M. , Giehl, E. L. H. , Lovato, P. E. , Vaz‐de‐Mello, F. Z. , Agudelo, M. B. , & Hernández, M. I. M. (2020). Dung beetles and the conservation of diversity in an agricultural landscape with maize fields and Atlantic Forest remnants. Acta Oecologica, 107, 103598. 10.1016/j.actao.2020.103598

[ece39831-bib-0002] Alves, V. M. , & Hernández, M. I. M. (2017). Morphometric modifications in Canthon quinquemaculatus Castelnau 1840 (Coleoptera: Scarabaeinae): Sublethal effects of transgenic maize? Insects, 8, 115. 10.3390/insects8040115 29065452 PMC5746798

[ece39831-bib-0003] Amat‐Garcia, G. , Lopera‐Toro, A. , & Amézquita‐Melo, S. J. (1997). Patrones de distribución de escarabajos coprófagos (Coleoptera: Scarabaeidae) en relicto del bosque altoandino, cordillera oriental de Colombia. Caldasia, 1–2, 113–126. 10.21829/azm.1999.76761703

[ece39831-bib-0004] Amézquita, S. , Forsyth, A. , Lopera, A. , & Camacho, A. (1999). Comparación de la composición y riqueza de especies de escarabajos coprófagos (Coleoptera: Scarabaeidae) en remanentes de bosque de la orinoquia colombiana. Acta Zoológica Mexicana (N.S.), 76, 113–126. 10.21829/azm.1999.76761703

[ece39831-bib-0005] Barragán, F. , Moreno, C. E. , Escobar, F. , Bueno‐Villegas, J. , & Halffter, G. (2014). The impact of grazing on dung beetle diversity depends on both biogeographical and ecological context. Journal of Biogeography, 41, 1991–2002. 10.1111/jbi.12351

[ece39831-bib-0006] Bendix, J. , Homeier, J. , Ortiz, E. C. , Emck, P. , Breckle, S. W. , Richter, M. , & Beck, E. (2006). Seasonality of weather and tree phenology in a tropical evergreen mountain rain forest. International Journal of Biometeorology, 50, 370–384. 10.1007/s00484-006-0029-8 16598482

[ece39831-bib-0007] Blanckenhorn, W. U. (2005). Behavioral causes and consequences of sexual size dimorphism. Ethology, 111, 977–1016. 10.1111/j.1439-0310.2005.01147.x

[ece39831-bib-0008] Burmeister, H. (1846). Genera quaedam insectorum. Iconibus illustravit et descripsit. Vol. 1. Berolini sumtibus A. Burmeister 1838–1846, unpaginated.

[ece39831-bib-0009] Cadotte, M. W. , Carscadden, K. , & Mirotchnick, N. (2011). Beyond species: Functional diversity and the maintenance of ecological processes and services. Journal of Applied Ecology, 48, 1079–1087. 10.1111/j.1365-2664.2011.02048.x

[ece39831-bib-0010] Carrión‐Paladines, V. , Fries, A. , Muñoz, A. , Castillo, E. , García‐Ruiz, R. , & Marín‐Armijos, D. (2021). Effects of land‐use change on the community structure of the dung beetle (Scarabaeinae) in an altered ecosystem in southern Ecuador. Insects, 12, 306. 10.3390/insects12040306 33808282 PMC8066223

[ece39831-bib-0011] Carter, A. W. , & Sheldon, K. S. (2020). Life stages differ in plasticity to temperature fluctuations and uniquely contribute to adult phenotype in Onthophagus taurus dung beetles. The Journal of Experimental Biology, 223, jeb227884. 10.1242/jeb.227884 32917819

[ece39831-bib-0012] Chamorro, W. , Marín‐Armijos, D. , Asenjo, A. , & Vaz‐de‐Mello, F. Z. (2019). Scarabaeinae dung beetles from Ecuador: A catalog, nomenclatural acts, and distribution records. Zookeys, 826, 1–343. 10.3897/zookeys.826.26488 30858752 PMC6405737

[ece39831-bib-0013] Chamorro, W. , Marín‐Armijos, D. , Granda, V. , & Vaz‐de‐Mello, F. Z. (2018). Listado de especies y clave de géneros y subgéneros de escarabajos estercoleros (Coleoptera: Scarabaeidae: Scarabaeinae) presentes y presuntos para Ecuador. Revista Colombiana De Entomología, 44, 72–100. 10.25100/socolen.v44i1.6545

[ece39831-bib-0014] Daoudi, L. , Chavanon, G. , Taybi, A. F. , & Mabrouki, Y. (2022). Composition and phenology of the beetle community (Coleoptera: Scarabaeoidea, Staphylinidae, Histeridae, Hydrophilidae) associated to dung of equines in an arid environment. Ann De La Société Entomologique De France N S, 1–10, 155–164. 10.1080/00379271.2022.2050470

[ece39831-bib-0015] deCastro‐Arrazola, I. , Hortal, J. , Noriega, J. A. , & Sánchez‐Piñero, F. (2020). Assessing the functional relationship between dung beetle traits and dung removal, burial, and seedling emergence. Ecology, 101, e03138. 10.1002/ecy.3138 32691865

[ece39831-bib-0016] Edwards, F. A. , Edwards, D. P. , Larsen, T. H. , Hsu, W. W. , Benedick, S. , Chung, A. , Vun Khen, C. , Wilcove, D. S. , & Hamer, K. C. (2014). Does logging and forest conversion to oil palm agriculture alter functional diversity in a biodiversity hotspot? Animal Conservation, 17, 163–173. 10.1111/acv.12074 25821399 PMC4372061

[ece39831-bib-0017] Gagic, V. , Bartomeus, I. , Jonsson, T. , Taylor, A. , Winqvist, C. , Fischer, C. , Slade, E. M. , Steffan‐Dewenter, I. , Emmerson, M. , Potts, S. G. , Tscharntke, T. , Weisser, W. , & Bommarco, R. (2015). Functional identity and diversity of animals predict ecosystem functioning better than species‐based indices. Proceedings of the Royal Society B: Biological Sciences, 282, 20142620. 10.1098/rspb.2014.2620 PMC4309003 25567651

[ece39831-bib-0018] Gardner, T. A. , Hernández, M. I. M. , Barlow, J. , & Peres, C. A. (2008). Understanding the biodiversity consequences of habitat change: The value of secondary and plantation forests for neotropical dung beetles. Journal of Applied Ecology, 45, 883–893. 10.1111/j.1365-2664.2008.01454.x

[ece39831-bib-0019] Gómez, V. C. G. , Verdú, J. R. , Gómez‐Cifuentes, A. , Vaz‐de‐Mello, F. Z. , & Zurita, G. A. (2018). Influence of land use on the trophic niche overlap of dung beetles in the semideciduous Atlantic forest of Argentina. Insect Conserv Diver, 11, 554–564. 10.1111/icad.12299

[ece39831-bib-0020] Goolsby, J. A. , Singh, N. K. , Thomas, D. B., Jr. , Ortega‐S, A., Jr. , Hewitt, D. G. , Campbell, T. A. , & de Leon, A. P. (2017). Comparison of chemical attractants against dung beetles and application for rangeland and animal health. South West Entomology, 42, 339–346. 10.3958/059.042.0203

[ece39831-bib-0021] Hanski, I. , & Cambefort, Y. (1991). Dung Beetle Ecology (pp. 350–365). Princeton University Press. 10.1515/9781400862092.350

[ece39831-bib-0022] Hernández, M. I. M. , Monteiro, L. R. , & Favila, M. E. (2011). The role of body size and shape in understanding competitive interactions within a Community of Neotropical Dung Beetles. Journal of Insect Science, 11, 1–14. 10.1673/031.011.0113 21526928 PMC3281377

[ece39831-bib-0023] Hu, Y. , Linz, D. M. , Parker, E. S. , Schwab, D. B. , Casasa, S. , Macagno, A. L. M. , & Moczek, A. P. (2020). Developmental bias in horned dung beetles and its contributions to innovation, adaptation, and resilience. Evolution & Development, 22, 165–180. 10.1111/ede.12310 31475451

[ece39831-bib-0024] Larsen, T. H. , & Forsyth, A. (2005). Trap spacing and transect design for dung beetle biodiversity Studies1. Biotropica, 37, 322–325. 10.1111/j.1744-7429.2005.00042.x

[ece39831-bib-0025] Larsen, T. H. , Lopera, A. , Forsyth, A. , & Gnier, F. (2009). From coprophagy to predation: A dung beetle that kills millipedes. Biology Letters, 5, 152–155. 10.1098/rsbl.2008.0654 19158030 PMC2665820

[ece39831-bib-0026] Larsen, T. H. , Williams, N. M. , & Kremen, C. (2005). Extinction order and altered community structure rapidly disrupt ecosystem functioning. Ecology Letters, 8, 538–547. 10.1111/j.1461-0248.2005.00749.x 21352458

[ece39831-bib-0027] Luederwaldt, H. (1929). As espécies brasileiras do gênero. Pinotus. Revista do Museu Paulista, 16, 603–776.

[ece39831-bib-0028] Lobo, J. M. , & Cuesta, E. (2021). Seasonal variation in the diel activity of a dung beetle assemblage. PeerJ, 9, e11786. 10.7717/peerj.11786 34306833 PMC8280883

[ece39831-bib-0029] Macagno, A. L. M. , Moczek, A. P. , & Pizzo, A. (2016). Rapid divergence of nesting depth and digging appendages among tunneling dung beetle populations and species. The American Naturalist, 187, E143–E151. 10.1086/685776 27105002

[ece39831-bib-0030] Mamantov, M. A. , & Sheldon, K. S. (2021). Behavioural responses to warming differentially impact survival in introduced and native dung beetles. The Journal of Animal Ecology, 90, 273–281. 10.1111/1365-2656.13366 33037612

[ece39831-bib-0031] Martín, C. M. , Guanuco, A. D. V. , Cortez, V. , & Verdú, J. R. (2021). First observation on the predation of a non‐arthropod species by a dung beetle species: The case of Canthon chalybaeus and the snail Bulimulus apodemetes. PLoS One, 16, e0258396. 10.1371/journal.pone.0258396 34644349 PMC8513830

[ece39831-bib-0032] Martínez, I. M. , Dellacasa, M. , Lumaret, J. P. , & Dellacasa, G. (2022). Phenology and reproductive cycles in Mexican aphodiine dung beetles (Coleoptera: Scarabaeidae: Aphodiinae: Aphodiini). Annales De La Société Entomologique De France NS, 58, 173–185. 10.1080/00379271.2022.2060859

[ece39831-bib-0033] Martínez, A. (1951). La invalidez del nombre genérico Pinotus Erichson y dos nuevas sinonímias (Col. Scarab.). Anales de La Sociedad Cientifica Argentina, 152, 138–142.

[ece39831-bib-0034] Menéndez, R. , González‐Megías, A. , Jay‐Robert, P. , & Marquéz‐Ferrando, R. (2014). Climate change and elevational range shifts: Evidence from dung beetles in two European mountain ranges. Global Ecology and Biogeography, 23, 646–657. 10.1111/geb.12142

[ece39831-bib-0035] Milotić, T. , Baltzinger, C. , Eichberg, C. , Eycott, A. E. , Heurich, M. , Müller, J. , Noriega, J. A. , Menendez, R. , Stadler, J. , Ádám, R. , Bargmann, T. , Bilger, I. , Buse, J. , Calatayud, J. , Ciubuc, C. , Boros, G. , Jay‐Robert, P. , Kruus, M. , Merivee, E. , … Hoffmann, M. (2019). Functionally richer communities improve ecosystem functioning: Dung removal and secondary seed dispersal by dung beetles in the Western Palaearctic. Journal of Biogeography, 46, 70–82. 10.1111/jbi.13452

[ece39831-bib-0036] Monteiro, B. , de Farias, P. M. , & Arellano, L. (2020). Dung removal by dichotomius (Luederwaldtinia) sericeus (Harold, 1867) (Coleoptera: Scarabaeidae: Scarabaeinae) in a laboratory setting, with behavioral notes. Coleopt Bulletin, 74, 726–730. 10.1649/0010-065x-74.4.726

[ece39831-bib-0037] Moser, G. , Hertel, D. , & Leuschner, C. (2007). Altitudinal change in LAI and stand leaf biomass in tropical montane forests: A transect study in Ecuador and a pan‐tropical meta‐analysis. Ecosystems, 10, 924–935. 10.1007/s10021-007-9063-6

[ece39831-bib-0038] Nichols, E. , Spector, S. , Louzada, J. , Larsen, T. , Amezquita, S. , Favila, M. E. , & Network, T. S. R. (2008). Ecological functions and ecosystem services provided by Scarabaeinae dung beetles. Biological Conservation, 141, 1461–1474. 10.1016/j.biocon.2008.04.011

[ece39831-bib-0039] Nunes, C. A. , Barlow, J. , França, F. , Berenguer, E. , Solar, R. R. C. , Louzada, J. , Leitão, R. P. , Maia, L. F. , Oliveira, V. H. F. , Braga, R. F. , Vaz‐de‐Mello, F. Z. , & Sayer, E. J. (2021). Functional redundancy of Amazonian dung beetles confers community‐level resistance to primary forest disturbance. Biotropica, 53, 1510–1521. 10.1111/btp.12998

[ece39831-bib-0040] Nunes, R. V. , & Vaz‐de‐Mello, F. Z. (2020). Taxonomic revision of dichotomius (Cephagonus) Luederwaldt 1929 and the taxonomic status of remaining dichotomius hope 1838 subgenera (Coleoptera: Scarabaeidae: Scarabaeinae: Dichotomiini). Journal of Natural History, 53, 2231–2351. 10.1080/00222933.2019.1692088

[ece39831-bib-0041] Oksanen, J. , Blanchet, F. G. , Friendly, M. , Kindt, R. , Legendre, P. , McGlinn, D. , O'Hara, R. B. , Solymos, P. , Stevens, H. M. , Szoecs, E. , Wagne, H. , Barbour, M. , Bedward, M. , Bolker, B. , Borcard, D. , Carvalho, G. , Chirico, M. , De Caceres, M. , Durand, S. , … Weedon, J. (2019). Vegan: Community ecology package. R Package Version, 2, 5–6. Available at:. https://CRAN.R‐project.org/package=vegan

[ece39831-bib-0042] Ortega‐Martínez, I. J. , Moreno, C. E. , Arellano, L. , Castellanos, I. , Rosas, F. , & Ríos‐Díaz, C. L. (2021). The relationship between dung beetle diversity and manure removal in forest and sheep grazed grasslands. Community Ecology, 1–11, 135–145. 10.1007/s42974-021-00043-w

[ece39831-bib-0043] Pardo‐Diaz, C. , Toro, A. L. , Tovar, S. A. P. , Sarmiento‐Garcés, R. , Herrera, M. S. , & Salazar, C. (2019). Taxonomic reassessment of the genus Dichotomius (Coleoptera: Scarabaeinae) through integrative taxonomy. PeerJ, 7, e7332. 10.7717/peerj.7332 31404430 PMC6686840

[ece39831-bib-0044] Raine, E. H. , Gray, C. L. , Mann, D. J. , & Slade, E. M. (2018). Tropical dung beetle morphological traits predict functional traits and show intraspecific differences across land uses. Ecology and Evolution, 8, 8686–8696. 10.1002/ece3.4218 30271537 PMC6157683

[ece39831-bib-0045] R‐Core‐Team . (2019). R: A language and environment for statistical computing. R Foundation for Statistical Computing, . Available at. https://www.R‐project.org/

[ece39831-bib-0046] Rossini, M. , & Vaz‐de‐Mello, F. Z. (2020). Taxonomic review of the dichotomius mamillatus group (Coleoptera: Scarabaeidae), with a description of a new species, Dichotomius (Dichotomius) gandinii sp. nov., from western Amazonia. Austral Entomol, 59, 52–73. 10.1111/aen.12443

[ece39831-bib-0047] Salomão, R. P. , Cerqueira, L. V. M. P. , Gomes, A. d. A. C. , González‐Tokman, D. , Maia, A. C. D. , & Iannuzzi, L. (2022). Dung or carrion? Sex and age determine resource attraction in dung beetles. Ecological Entomology, 47, 52–62. 10.1111/een.13090

[ece39831-bib-0048] Sarmiento‐Garcés, R. , & Amat‐García, G. (2009a). Escarabajos del género Dichotomius Hope 1838 (Scarabaeidae: Scarabaeinae) en la amazonía colombiana. Revista de la Academia Colombiana de Ciencias, 127, 285–296.

[ece39831-bib-0049] Sarmiento‐Garcés, R. , & Amat‐García, G. (2009b). Escarabajos del género dichotomius hope 1838 (Scarabaeidae: Scarbaeinae) en la Amazonía colombiana. Revista de la Academia Colombiana de Ciencias, 127, 285–296.

[ece39831-bib-0050] Schoolmeesters, P. (2021). Scarabs: World Scarabaeidae database (version Jan 2018). In Y. Roskov , L. Abucay , T. Orrell , D. Nicolson , N. Bailly , P. M. Kirk , T. Bourgoin , R. E. DeWalt , W. Decock , A. De Wever , N. E. van , J. Zarucchi , & L. Penev (Eds.), Species 2000 & ITIS catalogue of life, 2018 annual checklist. Species 2000: Naturalis. ISSN 2405‐884X. Accessed June 22, 2021. Digital resource at. www.catalogueoflife.org/annual‐checklist/2018.”>www.catalogueoflife.org/annual‐checklist/2018, www.catalogueoflife.org/annual‐checklist/2018

[ece39831-bib-0051] Seibold, S. , Gossner, M. M. , Simons, N. K. , Blüthgen, N. , Müller, J. , Ambarlı, D. , Ammer, C. , Bauhus, J. , Fischer, M. , Habel, J. C. , Linsenmair, K. E. , Nauss, T. , Penone, C. , Prati, D. , Schall, P. , Schulze, E. D. , Vogt, J. , Wöllauer, S. , & Weisser, W. W. (2019). Arthropod decline in grasslands and forests is associated with landscape‐level drivers. Nature, 574, 671–674. 10.1038/s41586-019-1684-3 31666721

[ece39831-bib-0052] Shahabuddin , Hidayat, P. , Manuwoto, S. , Noerdjito, W. A. , Tscharntke, T. , & Schulze, C. H. (2010). Diversity and body size of dung beetles attracted to different dung types along a tropical land‐use gradient in Sulawesi, Indonesia. Journal of Tropical Ecology, 26, 53–65. 10.1017/s0266467409990423

[ece39831-bib-0053] Slade, E. M. , Mann, D. J. , Villanueva, J. F. , & Lewis, O. T. (2007). Experimental evidence for the effects of dung beetle functional group richness and composition on ecosystem function in a tropical forest. The Journal of Animal Ecology, 76, 1094–1104. 10.1111/j.1365-2656.2007.01296.x 17922706

[ece39831-bib-0054] Sneed, E. D. , & Folk, R. L. (1958). Pebbles in the lower Colorado River, Texas a study in particle morphogenesis. The Journal of Geology, 66, 114–150. 10.1086/626490

[ece39831-bib-0055] Soto, C. S. , Giombini, M. I. , Gómez, V. C. G. , & Zurita, G. A. (2019). Phenotypic differentiation in a resilient dung beetle species induced by forest conversion into cattle pastures. Evolutionary Ecology, 33, 385–402. 10.1007/s10682-019-09987-y

[ece39831-bib-0056] Stanbrook, R. A. , Harris, W. E. , Wheater, C. P. , & Jones, M. (2021). Evidence of phenotypic plasticity along an altitudinal gradient in the dung beetle Onthophagus proteus. PeerJ, 9, e10798. 10.7717/peerj.10798 33665014 PMC7912602

[ece39831-bib-0057] Stillwell, R. C. , Blanckenhorn, W. U. , Teder, T. , Davidowitz, G. , & Fox, C. W. (2010). Sex differences in phenotypic plasticity affect variation in sexual size dimorphism in insects: From physiology to evolution. Annual Review of Entomology, 55, 227–245. 10.1146/annurev-ento-112408-085500 PMC4760685 19728836

[ece39831-bib-0058] Teder, T. , & Tammaru, T. (2005). Sexual size dimorphism within species increases with body size in insects. Oikos, 108, 321–334. 10.1111/j.0030-1299.2005.13609.x

[ece39831-bib-0059] Thormann, B. , Ahrens, D. , Espinosa, C. I. , Marín‐Armijos, D. , Wagner, T. , Wägele, J. W. , & Peters, M. K. (2018). Small‐scale topography modulates elevational α‐, β‐ and γ‐diversity of Andean leaf beetles. Oecologia, 92, 699–611. 10.1007/s00442-018-4108-4 29523951

[ece39831-bib-0060] Vaz‐de‐Mello, F. , Larsen, T. , Silva, F. , Gill, B. , Spector, S. , & Favila, M. (2014). Dichotomius problematicus. Dichotomius Problematicus. https://www.iucnredlist.org [Accessed June 28, 2021].

[ece39831-bib-0061] Venables, W. N. , & Ripley, B. D. (2002). Modern applied statistics with S (4th ed.). Springer Available at:. www.stats.ox.ac.uk/pub/MASS4

[ece39831-bib-0062] Verdú, J. R. , & Galante, E. (2002). Climatic stress, food availability and human activity as determinants of endemism patterns in the Mediterranean region: The case of dung beetles (Coleoptera, Scarabaeoidea) in the Iberian Peninsula. Diversity and Distributions, 8, 259–274.

