# Supplementary material for: Morphometric changes on dung beetle Dichotomius problematicus (Coleoptera: Scarabaeidae: Scarabaeinae) related to conversion of forest into grassland: A case of study in the Ecuadorian Amazonia
Source: Ecol Evol. 2023 Feb 17;13(2):e9831. doi: 10.1002/ece3.9831 (PMC9937892; doi:10.1002/ece3.9831)
Supplement: Supplementary file 1 — Table S1. [file ECE3-13-e9831-s001.docx]

**Appendix**

**Table 1.** Individuals measured per habitat (47 in forest and 222 in grassland). According to 13 morphological traits: head width (HW), head length (HL), pronotum width (PW), pronotum length (PL), pronotum high (PH), elytra length (EL), protibia width (pTW), protibia length (pTL), metatibia length (mTL), elytra width (I), body thickness (S), total length (L) and sphericity (Sph) according to: a) habitat (forest and grassland) and b) sexual dimorphism (female and male).

| **Ind_ID** | **Habitat** | **EL** | **HL** | **HW** | **mTL** | **PH** | **PL** | **pTL** | **pTW** | **PW** | **I** | **L** | **S** | **Sexual**  **dimorphism** | **Sph** |
| --- | --- | --- | --- | --- | --- | --- | --- | --- | --- | --- | --- | --- | --- | --- | --- |
| 1 | Grassland | 7.30 | 4.00 | 5.80 | 3.90 | 4.30 | 5.00 | 3.60 | 1.30 | 8.60 | 8.90 | 16.30 | 6.40 | Male | 0.09 |
| 2 | Grassland | 7.50 | 4.00 | 5.90 | 4.10 | 5.60 | 5.70 | 3.90 | 1.00 | 9.40 | 9.40 | 17.20 | 7.50 | Male | 0.12 |
| 3 | Grassland | 8.30 | 4.20 | 6.20 | 4.10 | 5.00 | 5.10 | 4.00 | 1.50 | 9.60 | 9.00 | 17.60 | 6.90 | Female | 0.10 |
| 4 | Grassland | 7.90 | 3.50 | 6.00 | 4.00 | 5.40 | 5.30 | 3.70 | 1.60 | 9.00 | 9.60 | 16.70 | 7.20 | Male | 0.11 |
| 5 | Grassland | 7.20 | 3.60 | 5.40 | 4.00 | 4.50 | 4.90 | 3.50 | 1.50 | 7.90 | 9.00 | 15.70 | 6.80 | Female | 0.11 |
| 6 | Grassland | 7.00 | 4.00 | 6.20 | 4.20 | 4.90 | 5.60 | 4.30 | 1.60 | 9.50 | 9.80 | 16.60 | 7.20 | Male | 0.11 |
| 7 | Grassland | 8.00 | 3.60 | 5.80 | 3.90 | 6.50 | 5.30 | 3.50 | 1.40 | 8.70 | 9.20 | 16.90 | 7.00 | Male | 0.11 |
| 8 | Grassland | 8.30 | 4.00 | 6.10 | 4.50 | 4.60 | 5.40 | 4.30 | 1.90 | 9.20 | 9.70 | 17.70 | 7.40 | Female | 0.11 |
| 9 | Grassland | 8.00 | 3.50 | 5.50 | 3.60 | 4.50 | 4.60 | 3.50 | 1.30 | 8.10 | 8.70 | 16.10 | 6.50 | Male | 0.10 |
| 10 | Grassland | 7.10 | 3.30 | 5.10 | 3.80 | 3.60 | 3.90 | 3.00 | 1.40 | 7.60 | 8.40 | 14.30 | 6.40 | Female | 0.11 |
| 11 | Grassland | 7.20 | 4.30 | 6.10 | 4.20 | 5.60 | 6.00 | 4.00 | 1.30 | 9.20 | 9.80 | 17.50 | 7.50 | Male | 0.11 |
| 12 | Grassland | 8.40 | 3.30 | 6.30 | 4.00 | 5.50 | 6.00 | 4.10 | 1.40 | 9.40 | 9.70 | 17.70 | 7.50 | Male | 0.11 |
| 13 | Grassland | 7.60 | 3.60 | 5.60 | 3.40 | 5.20 | 5.00 | 3.40 | 1.20 | 8.00 | 8.90 | 16.20 | 6.80 | Male | 0.11 |
| 14 | Grassland | 8.90 | 3.30 | 6.10 | 4.00 | 5.40 | 5.60 | 3.90 | 1.50 | 9.60 | 10.13 | 17.80 | 7.90 | Male | 0.12 |
| 15 | Grassland | 8.30 | 3.60 | 5.60 | 4.10 | 4.60 | 4.70 | 3.70 | 1.60 | 8.40 | 9.40 | 16.60 | 7.40 | Female | 0.12 |
| 16 | Grassland | 7.40 | 3.60 | 6.10 | 4.10 | 5.80 | 5.90 | 4.00 | 1.40 | 9.30 | 9.40 | 16.90 | 7.20 | Male | 0.11 |
| 17 | Grassland | 8.10 | 3.30 | 6.00 | 4.00 | 4.40 | 5.00 | 4.00 | 1.50 | 9.10 | 9.20 | 16.40 | 7.00 | Male | 0.11 |
| 18 | Grassland | 8.50 | 3.30 | 6.00 | 4.10 | 5.50 | 5.40 | 3.80 | 1.50 | 9.00 | 9.90 | 17.20 | 7.70 | Female | 0.12 |
| 19 | Grassland | 8.60 | 3.40 | 6.10 | 4.00 | 5.20 | 5.10 | 3.80 | 1.70 | 9.10 | 9.60 | 17.10 | 7.50 | Female | 0.11 |
| 20 | Grassland | 7.90 | 3.30 | 6.20 | 4.00 | 5.20 | 5.80 | 3.70 | 1.30 | 9.50 | 10.13 | 17.00 | 7.80 | Male | 0.12 |
| 21 | Grassland | 8.80 | 3.50 | 6.30 | 4.40 | 6.20 | 5.70 | 4.00 | 1.60 | 9.70 | 10.00 | 18.00 | 7.90 | Male | 0.12 |
| 22 | Grassland | 7.70 | 3.70 | 6.10 | 4.10 | 5.60 | 5.40 | 4.00 | 1.40 | 9.10 | 9.50 | 16.80 | 7.40 | Male | 0.11 |
| 23 | Grassland | 8.50 | 3.30 | 6.10 | 4.10 | 4.90 | 5.10 | 3.60 | 1.40 | 9.00 | 9.60 | 16.90 | 7.50 | Female | 0.12 |
| 24 | Grassland | 7.60 | 3.30 | 5.60 | 3.90 | 5.20 | 5.00 | 3.40 | 1.00 | 8.30 | 9.00 | 15.90 | 6.50 | Female | 0.10 |
| 25 | Grassland | 7.30 | 3.30 | 5.80 | 3.80 | 5.50 | 5.30 | 3.60 | 1.40 | 8.70 | 9.30 | 15.90 | 6.70 | Male | 0.10 |
| 26 | Grassland | 7.60 | 3.50 | 5.70 | 4.10 | 4.60 | 5.30 | 3.60 | 1.30 | 8.70 | 9.30 | 16.40 | 7.00 | Male | 0.11 |
| 27 | Grassland | 6.90 | 3.70 | 6.00 | 4.10 | 5.20 | 5.40 | 3.70 | 1.30 | 9.10 | 9.40 | 16.00 | 7.20 | Male | 0.11 |
| 28 | Grassland | 7.30 | 3.50 | 5.50 | 4.10 | 4.50 | 4.80 | 3.60 | 1.40 | 8.40 | 8.60 | 15.60 | 6.30 | Male | 0.10 |
| 29 | Grassland | 7.30 | 3.50 | 5.70 | 4.20 | 5.00 | 5.20 | 3.50 | 1.50 | 8.60 | 8.90 | 16.00 | 6.50 | Female | 0.10 |
| 30 | Grassland | 7.80 | 3.20 | 5.70 | 4.10 | 4.30 | 4.70 | 3.40 | 1.40 | 8.50 | 9.20 | 15.70 | 6.50 | Female | 0.10 |
| 31 | Grassland | 8.00 | 3.30 | 5.90 | 4.20 | 4.90 | 5.10 | 4.00 | 1.70 | 8.90 | 9.50 | 16.40 | 7.30 | Female | 0.11 |
| 32 | Grassland | 8.20 | 3.40 | 6.10 | 4.00 | 5.40 | 5.50 | 3.90 | 1.40 | 9.50 | 9.60 | 17.10 | 7.40 | Male | 0.11 |
| 33 | Grassland | 7.40 | 3.50 | 5.80 | 3.80 | 4.80 | 5.10 | 3.80 | 1.60 | 8.50 | 9.20 | 16.00 | 6.80 | Female | 0.10 |
| 34 | Grassland | 8.20 | 3.10 | 6.30 | 4.20 | 5.10 | 6.00 | 3.90 | 1.30 | 9.80 | 10.00 | 17.30 | 7.90 | Female | 0.12 |
| 35 | Grassland | 6.90 | 3.40 | 5.40 | 3.90 | 4.90 | 4.90 | 3.30 | 1.50 | 8.00 | 8.50 | 15.20 | 6.40 | Female | 0.11 |
| 36 | Grassland | 8.80 | 3.40 | 6.40 | 4.40 | 5.60 | 5.70 | 4.20 | 1.30 | 9.70 | 10.25 | 17.90 | 8.20 | Female | 0.12 |
| 37 | Grassland | 8.50 | 3.60 | 6.00 | 4.10 | 5.30 | 5.50 | 4.00 | 1.40 | 9.20 | 9.50 | 17.60 | 7.50 | Male | 0.11 |
| 38 | Grassland | 8.60 | 3.40 | 6.00 | 4.00 | 7.00 | 5.40 | 3.90 | 1.50 | 8.60 | 9.60 | 17.40 | 7.50 | Female | 0.11 |
| 39 | Grassland | 8.50 | 3.40 | 6.00 | 4.20 | 5.10 | 5.20 | 3.50 | 1.30 | 9.00 | 9.60 | 17.10 | 7.50 | Female | 0.11 |
| 40 | Grassland | 8.30 | 3.60 | 5.90 | 4.20 | 4.10 | 5.00 | 3.70 | 1.50 | 8.30 | 8.80 | 16.90 | 6.60 | Female | 0.10 |
| 41 | Grassland | 8.20 | 3.60 | 6.20 | 4.10 | 4.90 | 5.50 | 4.00 | 1.60 | 9.00 | 9.50 | 17.30 | 7.50 | Female | 0.11 |
| 42 | Grassland | 8.00 | 3.40 | 5.50 | 3.30 | 4.90 | 4.80 | 3.40 | 1.10 | 8.10 | 8.90 | 16.20 | 6.90 | Male | 0.11 |
| 43 | Grassland | 8.20 | 3.40 | 6.20 | 4.00 | 5.50 | 5.80 | 4.10 | 1.40 | 9.60 | 9.90 | 17.40 | 7.50 | Male | 0.11 |
| 44 | Grassland | 8.00 | 3.10 | 6.40 | 3.90 | 6.40 | 5.90 | 4.00 | 1.40 | 9.80 | 10.25 | 17.00 | 7.60 | Male | 0.11 |
| 45 | Grassland | 6.60 | 2.20 | 4.80 | 3.10 | 4.00 | 4.10 | 3.00 | 1.00 | 7.10 | 7.50 | 12.90 | 6.00 | Male | 0.12 |
| 46 | Grassland | 8.60 | 3.30 | 6.00 | 3.90 | 5.50 | 5.20 | 3.60 | 1.40 | 9.20 | 9.30 | 17.10 | 7.30 | Female | 0.11 |
| 47 | Grassland | 8.30 | 3.40 | 5.70 | 4.20 | 3.80 | 4.60 | 3.60 | 1.30 | 8.00 | 9.00 | 16.30 | 6.90 | Female | 0.11 |
| 48 | Grassland | 7.70 | 3.40 | 6.40 | 4.40 | 5.00 | 5.80 | 4.20 | 1.60 | 9.80 | 10.63 | 16.90 | 8.10 | Female | 0.12 |
| 49 | Grassland | 9.10 | 3.20 | 6.20 | 4.30 | 5.50 | 5.00 | 4.00 | 1.50 | 9.40 | 9.60 | 17.30 | 7.50 | Female | 0.11 |
| 50 | Grassland | 7.30 | 3.50 | 5.90 | 3.90 | 4.50 | 5.20 | 3.70 | 1.20 | 8.60 | 9.00 | 16.00 | 7.00 | Male | 0.11 |
| 51 | Grassland | 7.40 | 3.40 | 5.20 | 3.80 | 4.50 | 4.70 | 3.30 | 1.30 | 7.90 | 8.40 | 15.50 | 6.20 | Male | 0.10 |
| 52 | Grassland | 7.00 | 3.60 | 6.30 | 3.80 | 6.00 | 5.40 | 4.00 | 1.40 | 9.10 | 8.90 | 16.00 | 6.50 | Female | 0.10 |
| 53 | Grassland | 8.10 | 3.40 | 6.10 | 4.10 | 4.80 | 5.20 | 3.80 | 1.30 | 9.10 | 9.60 | 16.70 | 7.50 | Male | 0.12 |
| 54 | Grassland | 7.90 | 3.30 | 6.20 | 4.30 | 5.70 | 5.20 | 3.60 | 1.50 | 9.00 | 10.88 | 16.40 | 7.60 | Female | 0.11 |
| 55 | Grassland | 7.60 | 3.20 | 6.20 | 4.00 | 5.40 | 5.40 | 4.00 | 1.40 | 9.20 | 9.80 | 16.20 | 7.00 | Female | 0.10 |
| 56 | Grassland | 8.80 | 3.20 | 6.20 | 4.10 | 5.40 | 5.10 | 4.00 | 1.40 | 9.50 | 9.70 | 17.10 | 7.60 | Male | 0.12 |
| 57 | Grassland | 8.30 | 3.20 | 5.80 | 3.90 | 4.40 | 5.30 | 3.70 | 1.40 | 9.00 | 10.50 | 16.80 | 7.50 | Male | 0.11 |
| 58 | Grassland | 7.00 | 3.30 | 5.50 | 3.80 | 5.00 | 5.00 | 3.50 | 1.10 | 8.00 | 7.80 | 15.30 | 5.90 | Male | 0.10 |
| 59 | Grassland | 9.10 | 3.40 | 6.10 | 4.30 | 5.40 | 5.20 | 4.00 | 1.30 | 9.40 | 9.60 | 17.70 | 7.10 | Male | 0.10 |
| 60 | Grassland | 8.40 | 3.20 | 6.00 | 4.10 | 5.00 | 5.50 | 4.00 | 1.20 | 9.00 | 9.00 | 17.10 | 6.90 | Male | 0.10 |
| 61 | Grassland | 8.60 | 3.20 | 6.20 | 4.00 | 5.30 | 5.20 | 4.00 | 1.10 | 9.30 | 10.50 | 17.00 | 7.90 | Male | 0.12 |
| 62 | Grassland | 6.60 | 3.40 | 5.10 | 3.80 | 5.30 | 4.50 | 3.40 | 1.20 | 7.70 | 8.70 | 14.50 | 6.00 | Female | 0.10 |
| 63 | Grassland | 8.50 | 3.60 | 6.20 | 4.10 | 5.40 | 5.50 | 4.20 | 1.50 | 9.20 | 10.00 | 17.60 | 8.30 | Female | 0.13 |
| 64 | Grassland | 8.00 | 3.50 | 6.10 | 4.00 | 5.40 | 5.30 | 4.00 | 1.50 | 9.40 | 9.30 | 16.80 | 7.30 | Male | 0.11 |
| 65 | Grassland | 7.50 | 3.30 | 5.10 | 3.90 | 4.10 | 5.10 | 3.00 | 1.00 | 7.80 | 8.50 | 15.90 | 6.50 | Female | 0.10 |
| 66 | Grassland | 9.00 | 3.70 | 6.20 | 4.20 | 6.00 | 4.90 | 4.00 | 1.40 | 9.90 | 10.25 | 17.60 | 7.40 | Male | 0.10 |
| 67 | Grassland | 7.50 | 3.40 | 5.60 | 4.00 | 4.60 | 5.30 | 4.10 | 1.30 | 8.60 | 9.00 | 16.20 | 7.00 | Male | 0.11 |
| 68 | Grassland | 8.00 | 3.20 | 5.20 | 3.50 | 4.20 | 4.60 | 3.20 | 1.00 | 7.50 | 7.80 | 15.80 | 6.00 | Male | 0.10 |
| 69 | Grassland | 8.20 | 3.30 | 6.10 | 4.10 | 5.20 | 5.10 | 3.80 | 1.60 | 8.90 | 9.50 | 16.60 | 7.50 | Female | 0.12 |
| 70 | Grassland | 8.50 | 3.40 | 6.00 | 4.10 | 5.10 | 5.50 | 3.80 | 1.40 | 9.20 | 9.70 | 17.40 | 7.00 | Female | 0.10 |
| 71 | Grassland | 8.80 | 3.20 | 6.20 | 4.20 | 5.70 | 5.40 | 3.60 | 1.20 | 9.30 | 9.90 | 17.40 | 7.50 | Male | 0.11 |
| 72 | Grassland | 7.80 | 3.00 | 5.50 | 3.80 | 4.60 | 5.20 | 3.50 | 1.10 | 8.50 | 9.00 | 16.00 | 6.60 | Male | 0.10 |
| 73 | Grassland | 8.00 | 3.60 | 6.40 | 4.10 | 6.50 | 5.90 | 4.20 | 1.50 | 9.70 | 10.38 | 17.50 | 8.00 | Male | 0.12 |
| 74 | Grassland | 8.30 | 3.40 | 5.70 | 4.00 | 5.00 | 5.20 | 3.90 | 1.40 | 8.70 | 9.00 | 16.90 | 7.00 | Male | 0.11 |
| 75 | Grassland | 8.20 | 3.10 | 6.00 | 3.90 | 5.00 | 5.10 | 3.90 | 1.20 | 9.00 | 9.40 | 16.40 | 7.10 | Male | 0.11 |
| 76 | Grassland | 8.50 | 4.50 | 6.40 | 4.20 | 6.40 | 5.80 | 4.00 | 1.50 | 9.80 | 10.13 | 18.80 | 8.00 | Male | 0.11 |
| 77 | Grassland | 8.00 | 3.40 | 6.00 | 4.20 | 5.10 | 5.20 | 4.00 | 1.40 | 9.20 | 9.40 | 16.60 | 7.20 | Male | 0.11 |
| 78 | Grassland | 9.00 | 3.30 | 6.40 | 4.30 | 5.50 | 5.30 | 4.00 | 1.30 | 9.50 | 10.00 | 17.60 | 7.60 | Male | 0.11 |
| 79 | Grassland | 8.50 | 3.30 | 6.20 | 4.00 | 5.20 | 5.50 | 3.60 | 1.60 | 9.30 | 9.90 | 17.30 | 7.80 | Female | 0.12 |
| 80 | Grassland | 7.20 | 3.20 | 6.30 | 4.40 | 5.20 | 5.70 | 3.90 | 1.50 | 9.50 | 10.63 | 16.10 | 7.80 | Female | 0.12 |
| 81 | Grassland | 8.00 | 3.10 | 5.40 | 4.10 | 5.20 | 5.10 | 4.00 | 1.30 | 8.60 | 9.30 | 16.20 | 6.90 | Female | 0.11 |
| 82 | Grassland | 8.20 | 3.40 | 6.40 | 4.00 | 6.00 | 5.80 | 4.10 | 1.40 | 9.70 | 10.88 | 17.40 | 7.70 | Male | 0.10 |
| 83 | Grassland | 7.50 | 3.00 | 5.20 | 3.50 | 4.60 | 4.40 | 3.10 | 1.10 | 7.70 | 8.40 | 14.90 | 6.00 | Female | 0.10 |
| 84 | Grassland | 8.30 | 3.40 | 6.40 | 4.50 | 5.00 | 5.50 | 4.10 | 1.50 | 9.80 | 11.13 | 17.20 | 8.60 | Female | 0.13 |
| 85 | Grassland | 9.00 | 3.20 | 6.20 | 4.20 | 5.80 | 4.90 | 4.00 | 1.60 | 9.50 | 10.13 | 17.10 | 7.60 | Female | 0.11 |
| 86 | Grassland | 7.40 | 3.20 | 5.50 | 3.80 | 4.90 | 5.00 | 3.60 | 1.30 | 8.40 | 9.30 | 15.60 | 6.60 | Male | 0.10 |
| 87 | Grassland | 7.90 | 3.20 | 6.60 | 3.60 | 4.60 | 5.00 | 3.90 | 1.20 | 8.30 | 9.00 | 16.10 | 6.60 | Female | 0.10 |
| 88 | Grassland | 8.00 | 3.20 | 5.40 | 4.20 | 4.10 | 5.30 | 3.90 | 1.60 | 9.00 | 9.40 | 16.50 | 7.40 | Female | 0.12 |
| 89 | Grassland | 8.20 | 3.20 | 5.40 | 4.00 | 5.00 | 5.20 | 3.70 | 1.30 | 8.70 | 9.20 | 16.60 | 7.20 | Male | 0.11 |
| 90 | Grassland | 7.60 | 3.00 | 5.70 | 4.10 | 5.00 | 5.20 | 3.60 | 1.40 | 8.70 | 9.20 | 15.80 | 7.20 | Male | 0.12 |
| 91 | Grassland | 8.50 | 3.50 | 6.10 | 4.20 | 5.50 | 5.20 | 3.90 | 1.60 | 9.20 | 9.80 | 17.20 | 7.60 | Female | 0.11 |
| 92 | Grassland | 7.50 | 3.20 | 5.80 | 3.90 | 5.30 | 4.60 | 3.90 | 1.40 | 8.30 | 9.20 | 15.30 | 6.50 | Female | 0.10 |
| 93 | Grassland | 7.80 | 3.40 | 6.10 | 4.10 | 5.00 | 5.80 | 4.00 | 1.60 | 9.50 | 10.25 | 17.00 | 7.60 | Female | 0.11 |
| 94 | Grassland | 7.60 | 3.60 | 5.70 | 4.00 | 4.90 | 5.20 | 3.50 | 1.40 | 8.60 | 8.90 | 16.40 | 7.40 | Female | 0.13 |
| 95 | Grassland | 7.40 | 3.40 | 5.30 | 3.90 | 4.00 | 4.70 | 3.50 | 1.30 | 8.00 | 8.50 | 15.50 | 6.50 | Female | 0.11 |
| 96 | Grassland | 6.90 | 3.50 | 5.90 | 4.00 | 5.20 | 5.00 | 3.70 | 1.20 | 8.60 | 9.20 | 15.40 | 6.90 | Female | 0.11 |
| 97 | Grassland | 7.90 | 3.20 | 6.00 | 4.00 | 5.00 | 5.40 | 3.20 | 1.40 | 9.00 | 9.40 | 16.50 | 7.20 | Male | 0.11 |
| 98 | Grassland | 8.10 | 3.20 | 5.80 | 4.00 | 4.50 | 5.10 | 4.10 | 1.50 | 8.60 | 9.10 | 16.40 | 7.00 | Female | 0.11 |
| 99 | Grassland | 8.20 | 3.50 | 6.00 | 4.10 | 5.10 | 5.30 | 3.60 | 1.50 | 8.80 | 9.50 | 17.00 | 7.50 | Female | 0.12 |
| 100 | Grassland | 8.20 | 3.60 | 6.50 | 4.30 | 5.00 | 6.10 | 4.20 | 1.60 | 9.80 | 10.38 | 17.90 | 8.00 | Male | 0.11 |
| 101 | Grassland | 8.40 | 3.30 | 6.20 | 4.30 | 4.80 | 5.50 | 3.60 | 1.70 | 9.30 | 10.00 | 17.20 | 7.70 | Female | 0.11 |
| 102 | Grassland | 8.30 | 3.40 | 6.10 | 4.30 | 4.70 | 5.50 | 4.00 | 1.40 | 9.40 | 9.60 | 17.20 | 7.60 | Female | 0.12 |
| 103 | Grassland | 8.40 | 3.50 | 6.50 | 4.40 | 5.20 | 5.60 | 4.20 | 1.90 | 9.60 | 10.75 | 17.50 | 7.50 | Female | 0.10 |
| 104 | Grassland | 7.40 | 3.00 | 5.60 | 3.80 | 4.60 | 5.50 | 3.50 | 1.40 | 8.90 | 9.00 | 15.90 | 6.80 | Male | 0.11 |
| 105 | Grassland | 8.10 | 3.30 | 5.50 | 3.80 | 4.00 | 4.50 | 3.60 | 1.50 | 8.20 | 8.80 | 15.90 | 7.00 | Female | 0.12 |
| 106 | Grassland | 8.00 | 4.00 | 6.00 | 4.40 | 4.50 | 5.10 | 4.00 | 1.50 | 9.00 | 9.70 | 17.10 | 7.50 | Female | 0.11 |
| 107 | Grassland | 8.30 | 3.30 | 6.00 | 4.50 | 5.50 | 5.20 | 3.60 | 1.20 | 9.10 | 9.70 | 16.80 | 7.50 | Female | 0.12 |
| 108 | Grassland | 8.80 | 3.10 | 6.10 | 4.20 | 5.50 | 5.30 | 4.00 | 1.50 | 9.20 | 10.00 | 17.20 | 7.70 | Female | 0.11 |
| 109 | Grassland | 7.80 | 3.50 | 6.00 | 4.10 | 5.50 | 5.40 | 3.90 | 1.60 | 8.90 | 9.80 | 16.70 | 7.50 | Female | 0.11 |
| 110 | Grassland | 8.00 | 3.40 | 6.00 | 4.10 | 6.00 | 5.60 | 4.00 | 1.40 | 9.50 | 10.25 | 17.00 | 7.70 | Male | 0.11 |
| 111 | Grassland | 8.00 | 3.20 | 5.70 | 4.10 | 5.10 | 5.00 | 3.70 | 1.70 | 8.70 | 9.30 | 16.20 | 7.00 | Female | 0.11 |
| 112 | Grassland | 8.00 | 3.30 | 6.00 | 4.10 | 5.00 | 5.50 | 4.00 | 1.30 | 9.00 | 9.20 | 16.80 | 7.30 | Male | 0.11 |
| 113 | Grassland | 9.10 | 3.50 | 6.40 | 4.40 | 5.20 | 5.40 | 4.10 | 1.40 | 9.40 | 9.80 | 18.00 | 7.70 | Female | 0.11 |
| 114 | Grassland | 8.00 | 3.50 | 5.30 | 3.90 | 4.60 | 5.10 | 3.40 | 1.30 | 8.70 | 9.10 | 16.60 | 7.00 | Male | 0.11 |
| 115 | Grassland | 8.10 | 3.40 | 5.80 | 4.20 | 4.50 | 5.10 | 3.50 | 1.30 | 5.80 | 9.00 | 16.60 | 7.00 | Female | 0.11 |
| 116 | Grassland | 7.60 | 3.10 | 5.40 | 3.80 | 5.20 | 4.60 | 3.50 | 1.60 | 8.20 | 8.70 | 15.30 | 7.00 | Female | 0.12 |
| 117 | Grassland | 8.20 | 3.30 | 6.00 | 4.20 | 6.40 | 5.10 | 3.90 | 1.40 | 9.20 | 10.00 | 16.60 | 7.50 | Female | 0.11 |
| 118 | Grassland | 7.10 | 3.30 | 5.20 | 3.70 | 5.00 | 4.70 | 3.60 | 1.50 | 8.00 | 8.20 | 15.10 | 6.30 | Female | 0.11 |
| 119 | Grassland | 7.40 | 3.20 | 5.90 | 4.20 | 5.00 | 5.50 | 3.70 | 1.50 | 9.20 | 9.30 | 16.10 | 7.00 | Male | 0.11 |
| 120 | Grassland | 8.30 | 3.00 | 5.60 | 3.80 | 5.00 | 5.20 | 3.70 | 1.30 | 8.60 | 9.00 | 16.50 | 7.00 | Male | 0.11 |
| 121 | Grassland | 8.00 | 3.10 | 5.70 | 4.10 | 5.20 | 5.00 | 3.70 | 1.50 | 8.70 | 9.20 | 16.10 | 7.20 | Female | 0.12 |
| 122 | Grassland | 7.20 | 2.90 | 5.00 | 3.80 | 5.50 | 4.50 | 3.50 | 1.20 | 7.60 | 8.10 | 14.60 | 6.00 | Male | 0.10 |
| 123 | Grassland | 7.60 | 3.10 | 5.70 | 3.50 | 5.50 | 5.50 | 3.50 | 1.20 | 8.70 | 9.30 | 16.20 | 7.50 | Male | 0.12 |
| 124 | Grassland | 8.30 | 3.30 | 6.00 | 4.50 | 5.50 | 5.20 | 3.60 | 1.20 | 9.10 | 9.70 | 16.80 | 7.50 | Female | 0.12 |
| 125 | Grassland | 7.50 | 3.50 | 6.20 | 4.00 | 7.00 | 6.10 | 4.10 | 1.30 | 10.25 | 10.25 | 17.10 | 8.00 | Female | 0.12 |
| 126 | Grassland | 7.40 | 3.00 | 5.40 | 3.60 | 5.50 | 5.20 | 3.30 | 1.20 | 8.00 | 8.50 | 15.60 | 6.50 | Male | 0.11 |
| 127 | Grassland | 8.00 | 3.40 | 6.10 | 4.20 | 5.50 | 5.30 | 4.00 | 1.60 | 9.30 | 9.80 | 16.70 | 7.60 | Female | 0.12 |
| 128 | Grassland | 8.00 | 3.40 | 6.00 | 4.00 | 5.00 | 5.20 | 3.50 | 1.40 | 9.00 | 9.50 | 16.60 | 7.50 | Female | 0.12 |
| 129 | Grassland | 8.40 | 3.40 | 6.00 | 4.50 | 5.50 | 5.20 | 3.60 | 1.20 | 9.10 | 9.80 | 17.00 | 7.50 | Female | 0.11 |
| 130 | Grassland | 8.20 | 3.50 | 6.10 | 4.50 | 5.30 | 5.30 | 3.60 | 1.20 | 9.20 | 9.60 | 17.00 | 7.40 | Male | 0.11 |
| 131 | Grassland | 8.30 | 3.00 | 6.00 | 4.00 | 5.50 | 5.20 | 3.40 | 1.30 | 9.10 | 9.20 | 16.50 | 7.00 | Female | 0.11 |
| 132 | Grassland | 8.10 | 3.00 | 6.00 | 4.10 | 5.50 | 5.00 | 3.20 | 1.40 | 9.10 | 8.70 | 16.10 | 6.40 | Male | 0.10 |
| 133 | Grassland | 8.00 | 3.00 | 6.00 | 4.10 | 5.50 | 5.00 | 3.20 | 1.40 | 9.10 | 8.40 | 16.00 | 6.10 | Male | 0.09 |
| 134 | Grassland | 8.10 | 3.00 | 6.00 | 4.10 | 5.50 | 5.10 | 3.20 | 1.30 | 9.10 | 8.30 | 16.20 | 6.40 | Male | 0.10 |
| 135 | Grassland | 8.40 | 3.30 | 6.20 | 4.20 | 5.60 | 5.20 | 3.30 | 1.20 | 9.30 | 9.70 | 16.90 | 7.40 | Female | 0.11 |
| 136 | Grassland | 8.60 | 3.40 | 6.00 | 4.20 | 5.50 | 5.40 | 3.40 | 1.40 | 9.10 | 9.00 | 17.40 | 6.80 | Male | 0.10 |
| 137 | Grassland | 8.40 | 3.40 | 6.10 | 4.20 | 5.70 | 5.20 | 4.00 | 1.60 | 9.00 | 9.40 | 17.00 | 7.20 | Male | 0.11 |
| 138 | Grassland | 8.30 | 3.30 | 6.20 | 4.20 | 5.50 | 5.40 | 4.00 | 1.50 | 9.40 | 9.80 | 17.00 | 7.50 | Female | 0.11 |
| 139 | Grassland | 8.50 | 3.50 | 6.30 | 4.10 | 5.20 | 5.50 | 3.90 | 1.60 | 9.60 | 9.40 | 17.50 | 7.50 | Male | 0.11 |
| 140 | Grassland | 8.60 | 3.60 | 6.30 | 4.00 | 5.70 | 6.00 | 3.80 | 1.40 | 9.60 | 9.10 | 18.20 | 7.50 | Female | 0.11 |
| 141 | Grassland | 8.40 | 3.20 | 6.10 | 4.10 | 5.40 | 5.20 | 3.80 | 1.40 | 9.10 | 9.90 | 16.80 | 7.60 | Male | 0.12 |
| 142 | Grassland | 8.60 | 3.50 | 6.40 | 4.10 | 5.40 | 5.90 | 3.80 | 1.60 | 9.70 | 9.90 | 18.00 | 7.60 | Male | 0.11 |
| 143 | Grassland | 8.30 | 3.40 | 6.50 | 4.00 | 5.50 | 5.60 | 3.90 | 1.40 | 9.40 | 9.90 | 17.30 | 7.50 | Male | 0.11 |
| 144 | Grassland | 8.20 | 3.20 | 6.10 | 4.00 | 5.10 | 5.30 | 3.80 | 1.30 | 9.00 | 9.70 | 16.70 | 7.40 | Female | 0.11 |
| 145 | Grassland | 8.40 | 3.50 | 7.00 | 3.90 | 5.40 | 5.50 | 3.80 | 1.20 | 9.70 | 10.25 | 17.40 | 8.00 | Male | 0.12 |
| 146 | Grassland | 8.30 | 3.30 | 6.20 | 4.20 | 5.50 | 5.40 | 4.00 | 1.50 | 9.40 | 9.80 | 17.00 | 7.50 | Female | 0.11 |
| 147 | Grassland | 8.40 | 3.40 | 6.20 | 4.00 | 5.50 | 5.50 | 3.80 | 1.40 | 9.20 | 9.90 | 17.30 | 7.50 | Male | 0.11 |
| 148 | Grassland | 8.20 | 3.20 | 6.10 | 4.00 | 5.50 | 5.50 | 3.80 | 1.20 | 9.30 | 9.80 | 16.90 | 7.50 | Female | 0.11 |
| 149 | Grassland | 8.70 | 3.50 | 7.00 | 4.10 | 6.00 | 5.80 | 4.10 | 1.60 | 9.80 | 10.00 | 18.00 | 8.00 | Male | 0.12 |
| 150 | Grassland | 8.00 | 3.20 | 5.70 | 4.10 | 5.10 | 5.00 | 3.70 | 1.70 | 8.70 | 9.30 | 16.20 | 7.00 | Female | 0.11 |
| 151 | Grassland | 8.00 | 3.30 | 6.00 | 4.10 | 5.00 | 5.50 | 4.00 | 1.30 | 9.00 | 9.20 | 16.80 | 7.30 | Male | 0.11 |
| 152 | Grassland | 9.10 | 3.50 | 6.40 | 4.40 | 5.20 | 5.40 | 4.10 | 1.40 | 9.40 | 9.80 | 18.00 | 7.70 | Female | 0.11 |
| 153 | Grassland | 8.00 | 3.50 | 5.30 | 3.90 | 4.60 | 5.10 | 3.40 | 1.30 | 8.70 | 9.10 | 16.60 | 7.00 | Male | 0.11 |
| 154 | Grassland | 8.10 | 3.40 | 5.80 | 4.20 | 4.50 | 5.10 | 3.50 | 1.30 | 5.80 | 9.00 | 16.60 | 7.00 | Female | 0.11 |
| 155 | Grassland | 8.30 | 3.30 | 6.20 | 4.20 | 5.50 | 5.40 | 4.00 | 1.50 | 9.40 | 9.80 | 17.00 | 7.50 | Female | 0.11 |
| 156 | Grassland | 7.80 | 3.00 | 6.20 | 3.80 | 5.50 | 5.20 | 3.40 | 1.20 | 9.40 | 8.60 | 16.00 | 6.40 | Male | 0.10 |
| 157 | Grassland | 6.90 | 3.70 | 6.00 | 4.10 | 5.20 | 5.40 | 3.70 | 1.30 | 9.10 | 9.40 | 16.00 | 7.20 | Male | 0.11 |
| 158 | Grassland | 7.30 | 3.50 | 5.50 | 4.10 | 4.50 | 4.80 | 3.60 | 1.40 | 8.40 | 8.60 | 15.60 | 6.30 | Male | 0.10 |
| 159 | Grassland | 7.30 | 3.50 | 5.70 | 4.20 | 5.00 | 5.20 | 3.50 | 1.50 | 8.60 | 8.90 | 16.00 | 6.50 | Male | 0.10 |
| 160 | Grassland | 7.80 | 3.20 | 5.70 | 4.10 | 4.30 | 4.70 | 3.40 | 1.40 | 8.50 | 9.20 | 15.70 | 6.50 | Male | 0.10 |
| 161 | Grassland | 8.70 | 3.50 | 5.90 | 4.20 | 4.90 | 5.60 | 4.00 | 1.70 | 8.90 | 10.25 | 17.80 | 8.50 | Female | 0.13 |
| 162 | Grassland | 8.20 | 3.40 | 6.10 | 4.00 | 5.40 | 5.50 | 3.90 | 1.40 | 9.50 | 9.60 | 17.10 | 7.40 | Male | 0.11 |
| 163 | Grassland | 7.40 | 3.50 | 5.80 | 3.80 | 4.80 | 5.10 | 3.80 | 1.60 | 8.50 | 9.20 | 16.00 | 6.80 | Male | 0.10 |
| 164 | Grassland | 8.20 | 3.10 | 6.30 | 4.20 | 5.10 | 6.00 | 3.90 | 1.30 | 9.80 | 10.00 | 17.30 | 7.90 | Male | 0.12 |
| 165 | Grassland | 6.90 | 3.40 | 5.40 | 3.90 | 4.90 | 4.90 | 3.30 | 1.50 | 8.00 | 8.50 | 15.20 | 6.40 | Female | 0.11 |
| 166 | Grassland | 8.80 | 3.40 | 6.40 | 4.40 | 5.60 | 5.70 | 4.20 | 1.30 | 9.70 | 10.25 | 17.90 | 8.20 | Male | 0.12 |
| 167 | Grassland | 8.50 | 3.60 | 6.00 | 4.10 | 5.30 | 5.50 | 4.00 | 1.40 | 9.20 | 9.50 | 17.60 | 7.50 | Female | 0.11 |
| 168 | Grassland | 8.60 | 3.40 | 6.00 | 4.00 | 7.00 | 5.40 | 3.90 | 1.50 | 8.60 | 9.60 | 17.40 | 7.50 | Male | 0.11 |
| 169 | Grassland | 8.50 | 3.40 | 6.00 | 4.20 | 5.10 | 5.20 | 3.50 | 1.30 | 9.00 | 9.60 | 17.10 | 7.50 | Female | 0.11 |
| 170 | Grassland | 8.30 | 3.60 | 5.90 | 4.20 | 4.10 | 5.00 | 3.70 | 1.50 | 8.30 | 8.80 | 16.90 | 6.60 | Female | 0.10 |
| 171 | Grassland | 8.20 | 3.60 | 6.20 | 4.10 | 4.90 | 5.50 | 4.00 | 1.60 | 9.00 | 9.50 | 17.30 | 7.50 | Male | 0.11 |
| 172 | Grassland | 8.00 | 3.40 | 5.50 | 3.30 | 4.90 | 4.80 | 3.40 | 1.10 | 8.10 | 8.90 | 16.20 | 6.90 | Male | 0.11 |
| 173 | Grassland | 8.20 | 3.40 | 6.20 | 4.00 | 5.50 | 5.80 | 4.10 | 1.40 | 9.60 | 9.90 | 17.40 | 7.50 | Female | 0.11 |
| 174 | Grassland | 8.00 | 3.10 | 6.40 | 3.90 | 6.40 | 5.90 | 4.00 | 1.40 | 9.80 | 7.90 | 17.00 | 6.40 | Female | 0.10 |
| 175 | Grassland | 5.70 | 3.00 | 4.80 | 3.10 | 4.00 | 4.50 | 3.00 | 1.00 | 7.10 | 8.90 | 13.20 | 5.40 | Female | 0.08 |
| 176 | Grassland | 8.60 | 3.30 | 6.00 | 3.90 | 5.50 | 5.20 | 3.60 | 1.40 | 9.20 | 9.30 | 17.10 | 7.30 | Female | 0.11 |
| 177 | Grassland | 8.30 | 3.40 | 5.70 | 4.20 | 3.80 | 4.60 | 3.60 | 1.30 | 8.00 | 9.00 | 16.30 | 6.90 | Female | 0.11 |
| 178 | Grassland | 7.70 | 3.40 | 6.40 | 4.40 | 5.00 | 5.80 | 4.20 | 1.60 | 9.80 | 10.63 | 16.90 | 8.10 | Female | 0.12 |
| 179 | Grassland | 9.10 | 3.20 | 6.20 | 4.30 | 5.50 | 5.00 | 4.00 | 1.50 | 9.40 | 9.60 | 17.30 | 7.50 | Female | 0.11 |
| 180 | Grassland | 7.30 | 3.50 | 5.90 | 3.90 | 4.50 | 5.20 | 3.70 | 1.20 | 8.60 | 9.00 | 16.00 | 7.00 | Male | 0.11 |
| 181 | Grassland | 7.40 | 3.40 | 5.20 | 3.80 | 4.50 | 4.70 | 3.30 | 1.30 | 7.90 | 8.40 | 15.50 | 6.20 | Female | 0.10 |
| 182 | Grassland | 5.20 | 3.60 | 3.30 | 1.30 | 9.50 | 6.20 | 7.90 | 3.70 | 5.80 | 17.00 | 0.00 | 10.13 | Male | 0.03 |
| 183 | Grassland | 8.10 | 7.80 | 6.10 | 4.10 | 4.80 | 5.20 | 3.80 | 1.30 | 9.10 | 9.60 | 21.10 | 7.50 | Male | 0.09 |
| 184 | Grassland | 7.90 | 3.30 | 6.20 | 4.30 | 5.70 | 5.20 | 3.60 | 1.50 | 9.00 | 10.88 | 16.40 | 7.60 | Female | 0.11 |
| 185 | Grassland | 7.60 | 3.20 | 6.20 | 4.00 | 5.40 | 5.40 | 4.00 | 1.40 | 9.20 | 9.80 | 16.20 | 7.00 | Female | 0.10 |
| 186 | Grassland | 8.80 | 3.20 | 6.20 | 4.10 | 5.40 | 5.10 | 4.00 | 1.40 | 9.50 | 9.70 | 17.10 | 7.60 | Male | 0.12 |
| 187 | Grassland | 8.30 | 3.20 | 5.80 | 3.90 | 4.40 | 5.30 | 3.70 | 1.40 | 9.00 | 10.50 | 16.80 | 7.50 | Female | 0.11 |
| 188 | Grassland | 7.00 | 3.30 | 5.50 | 3.80 | 5.00 | 5.00 | 3.50 | 1.10 | 8.00 | 7.80 | 15.30 | 5.90 | Male | 0.10 |
| 189 | Grassland | 7.20 | 2.40 | 4.10 | 3.70 | 3.70 | 4.60 | 3.40 | 1.10 | 6.20 | 7.20 | 14.20 | 4.80 | Male | 0.08 |
| 190 | Grassland | 8.40 | 3.20 | 6.00 | 4.10 | 5.00 | 5.50 | 4.00 | 1.20 | 9.00 | 9.00 | 17.10 | 6.90 | Male | 0.10 |
| 191 | Grassland | 8.60 | 3.20 | 6.20 | 4.00 | 5.30 | 5.20 | 4.00 | 1.10 | 9.30 | 10.50 | 17.00 | 7.90 | Female | 0.12 |
| 192 | Grassland | 6.60 | 3.40 | 5.10 | 3.80 | 5.30 | 4.50 | 3.40 | 1.20 | 7.70 | 8.70 | 14.50 | 6.00 | Female | 0.10 |
| 193 | Grassland | 8.50 | 3.60 | 6.20 | 4.10 | 5.40 | 5.50 | 4.20 | 1.50 | 9.20 | 10.00 | 17.60 | 8.30 | Female | 0.13 |
| 194 | Grassland | 8.00 | 3.50 | 6.10 | 4.00 | 5.40 | 5.30 | 4.00 | 1.50 | 9.40 | 9.30 | 16.80 | 7.30 | Female | 0.11 |
| 195 | Grassland | 7.50 | 3.30 | 5.10 | 3.90 | 4.10 | 5.10 | 3.00 | 1.00 | 7.80 | 8.50 | 15.90 | 6.50 | Male | 0.10 |
| 196 | Grassland | 9.00 | 3.70 | 6.20 | 4.20 | 6.00 | 4.90 | 4.00 | 1.40 | 9.90 | 10.25 | 17.60 | 7.40 | Male | 0.10 |
| 197 | Grassland | 7.50 | 3.40 | 5.60 | 4.00 | 4.60 | 5.30 | 4.10 | 1.30 | 8.60 | 9.00 | 16.20 | 7.00 | Male | 0.11 |
| 198 | Grassland | 8.00 | 3.20 | 5.20 | 3.50 | 4.20 | 4.60 | 3.20 | 1.00 | 7.50 | 7.80 | 15.80 | 6.00 | Male | 0.10 |
| 199 | Grassland | 8.20 | 3.30 | 6.10 | 4.10 | 5.20 | 5.10 | 3.80 | 1.60 | 8.90 | 9.50 | 16.60 | 7.50 | Female | 0.12 |
| 200 | Grassland | 8.50 | 3.40 | 6.00 | 4.10 | 5.10 | 5.50 | 3.80 | 1.40 | 9.20 | 9.70 | 17.40 | 7.00 | Male | 0.10 |
| 201 | Grassland | 8.80 | 3.20 | 6.20 | 4.20 | 5.70 | 5.40 | 3.60 | 1.20 | 9.30 | 9.90 | 17.40 | 7.50 | Male | 0.11 |
| 202 | Grassland | 7.80 | 3.00 | 5.50 | 3.80 | 4.60 | 5.20 | 3.50 | 1.10 | 8.50 | 9.00 | 16.00 | 6.60 | Female | 0.10 |
| 203 | Grassland | 8.00 | 3.60 | 6.40 | 4.10 | 6.50 | 5.90 | 4.20 | 1.50 | 9.70 | 10.38 | 17.50 | 8.00 | Female | 0.12 |
| 204 | Grassland | 8.30 | 3.40 | 5.70 | 4.00 | 5.00 | 5.20 | 3.90 | 1.40 | 8.70 | 9.00 | 16.90 | 7.00 | Female | 0.11 |
| 205 | Grassland | 8.50 | 3.30 | 6.10 | 4.10 | 4.90 | 5.10 | 3.60 | 1.40 | 9.00 | 9.60 | 16.90 | 7.50 | Female | 0.12 |
| 206 | Grassland | 7.60 | 3.30 | 5.60 | 3.90 | 5.20 | 5.00 | 3.40 | 1.00 | 8.30 | 9.00 | 15.90 | 6.50 | Male | 0.10 |
| 207 | Grassland | 7.30 | 3.30 | 5.80 | 3.80 | 5.50 | 5.30 | 3.60 | 1.40 | 8.70 | 9.30 | 15.90 | 6.70 | Female | 0.10 |
| 208 | Grassland | 7.60 | 3.50 | 5.70 | 4.10 | 4.60 | 5.30 | 3.60 | 1.30 | 8.70 | 9.30 | 16.40 | 7.00 | Female | 0.11 |
| 209 | Grassland | 6.90 | 3.70 | 6.00 | 4.10 | 5.20 | 5.40 | 3.70 | 1.30 | 9.10 | 9.40 | 16.00 | 7.20 | Male | 0.11 |
| 210 | Grassland | 7.30 | 3.50 | 5.50 | 4.10 | 4.50 | 4.80 | 3.60 | 1.40 | 8.40 | 8.60 | 15.60 | 6.30 | Female | 0.10 |
| 211 | Grassland | 7.30 | 3.50 | 5.70 | 4.20 | 5.00 | 5.20 | 3.50 | 1.50 | 8.60 | 8.90 | 16.00 | 6.50 | Male | 0.10 |
| 212 | Grassland | 7.80 | 3.20 | 5.70 | 4.10 | 4.30 | 4.70 | 3.40 | 1.40 | 8.50 | 9.20 | 15.70 | 6.50 | Male | 0.10 |
| 213 | Grassland | 8.00 | 3.30 | 5.90 | 4.20 | 4.90 | 5.10 | 4.00 | 1.70 | 8.90 | 9.50 | 16.40 | 7.30 | Male | 0.11 |
| 214 | Grassland | 8.20 | 3.40 | 6.10 | 4.00 | 5.40 | 5.50 | 3.90 | 1.40 | 9.50 | 9.60 | 17.10 | 7.40 | Male | 0.11 |
| 215 | Grassland | 7.40 | 3.50 | 5.80 | 3.80 | 4.80 | 5.10 | 3.80 | 1.60 | 8.50 | 9.20 | 16.00 | 6.80 | Female | 0.10 |
| 216 | Grassland | 8.20 | 3.10 | 6.30 | 4.20 | 5.10 | 6.00 | 3.90 | 1.30 | 9.80 | 10.00 | 17.30 | 7.90 | Male | 0.12 |
| 217 | Grassland | 6.90 | 3.40 | 5.40 | 3.90 | 4.90 | 4.90 | 3.30 | 1.50 | 8.00 | 8.50 | 15.20 | 6.40 | Male | 0.11 |
| 218 | Grassland | 8.80 | 3.40 | 6.40 | 4.40 | 5.60 | 5.70 | 4.20 | 1.30 | 9.70 | 10.25 | 17.90 | 8.20 | Male | 0.12 |
| 219 | Grassland | 8.50 | 3.60 | 6.00 | 4.10 | 5.30 | 5.50 | 4.00 | 1.40 | 9.20 | 9.50 | 17.60 | 7.50 | Male | 0.11 |
| 220 | Grassland | 8.60 | 3.40 | 6.00 | 4.00 | 7.00 | 5.40 | 3.90 | 1.50 | 8.60 | 9.60 | 17.40 | 7.50 | Male | 0.11 |
| 221 | Grassland | 8.50 | 3.40 | 6.00 | 4.20 | 5.10 | 5.20 | 3.50 | 1.30 | 9.00 | 9.60 | 17.10 | 7.50 | Female | 0.11 |
| 222 | Grassland | 7.70 | 3.50 | 6.10 | 4.20 | 6.10 | 5.70 | 4.00 | 1.30 | 9.20 | 9.70 | 16.90 | 8.00 | Female | 0.13 |
| 223 | Forest | 8.30 | 3.90 | 5.50 | 3.50 | 3.40 | 4.40 | 4.10 | 1.10 | 4.00 | 9.00 | 16.60 | 5.50 | Female | 0.07 |
| 224 | Forest | 8.60 | 3.60 | 5.70 | 4.30 | 3.60 | 5.00 | 4.20 | 0.90 | 8.10 | 9.00 | 17.20 | 5.60 | Male | 0.07 |
| 225 | Forest | 9.80 | 3.00 | 5.40 | 4.20 | 3.50 | 4.60 | 3.80 | 1.20 | 8.50 | 9.20 | 17.40 | 5.60 | Male | 0.07 |
| 226 | Forest | 9.30 | 4.10 | 6.10 | 4.50 | 5.20 | 4.90 | 4.00 | 1.40 | 9.70 | 10.38 | 18.30 | 6.70 | Male | 0.08 |
| 227 | Forest | 9.00 | 3.60 | 5.80 | 4.00 | 3.70 | 4.70 | 3.20 | 1.20 | 8.60 | 9.80 | 17.30 | 6.00 | Female | 0.07 |
| 228 | Forest | 8.10 | 2.60 | 5.50 | 3.60 | 4.40 | 4.70 | 2.90 | 1.10 | 8.40 | 8.70 | 15.40 | 5.90 | Male | 0.09 |
| 229 | Forest | 7.10 | 3.70 | 5.30 | 4.00 | 4.50 | 4.50 | 3.10 | 1.20 | 8.10 | 8.80 | 15.30 | 5.60 | Female | 0.08 |
| 230 | Forest | 7.90 | 2.60 | 5.10 | 3.50 | 3.90 | 4.40 | 3.20 | 1.10 | 7.80 | 8.40 | 14.90 | 5.30 | Male | 0.07 |
| 231 | Forest | 9.10 | 5.00 | 6.00 | 4.00 | 6.20 | 5.20 | 3.00 | 1.30 | 8.50 | 9.10 | 19.30 | 6.40 | Female | 0.08 |
| 232 | Forest | 7.50 | 3.60 | 5.60 | 3.40 | 4.70 | 4.90 | 3.40 | 1.20 | 8.10 | 8.60 | 16.00 | 5.30 | Female | 0.07 |
| 233 | Forest | 9.10 | 3.70 | 5.80 | 6.20 | 4.00 | 4.90 | 5.60 | 2.70 | 9.00 | 9.80 | 17.70 | 5.80 | Female | 0.06 |
| 234 | Forest | 8.20 | 3.50 | 5.60 | 4.00 | 3.50 | 4.60 | 3.00 | 1.20 | 8.10 | 9.10 | 16.30 | 5.50 | Male | 0.07 |
| 235 | Forest | 9.20 | 5.00 | 5.70 | 4.10 | 3.60 | 5.00 | 3.60 | 1.40 | 9.10 | 10.63 | 19.20 | 6.20 | Male | 0.06 |
| 236 | Forest | 8.80 | 3.80 | 6.10 | 4.00 | 5.30 | 4.50 | 3.70 | 1.40 | 9.10 | 10.30 | 17.10 | 7.30 | Female | 0.10 |
| 237 | Forest | 8.10 | 4.20 | 5.10 | 3.70 | 4.80 | 4.80 | 3.40 | 1.50 | 8.50 | 9.20 | 17.10 | 6.80 | Female | 0.10 |
| 238 | Forest | 8.60 | 4.50 | 5.60 | 4.20 | 4.30 | 5.00 | 3.10 | 1.10 | 8.00 | 9.12 | 18.10 | 6.75 | Female | 0.09 |
| 239 | Forest | 9.00 | 4.10 | 6.00 | 4.10 | 5.00 | 5.40 | 3.50 | 1.40 | 8.50 | 10.50 | 18.50 | 6.10 | Female | 0.06 |
| 240 | Forest | 7.90 | 3.80 | 5.40 | 3.80 | 5.00 | 4.70 | 3.40 | 1.10 | 7.50 | 8.60 | 16.40 | 6.20 | Male | 0.09 |
| 241 | Forest | 9.20 | 3.90 | 5.40 | 4.10 | 5.90 | 5.00 | 3.10 | 1.40 | 8.10 | 9.80 | 18.10 | 6.60 | Male | 0.08 |
| 242 | Forest | 8.50 | 4.10 | 5.40 | 4.00 | 5.00 | 5.10 | 3.50 | 1.20 | 8.00 | 8.90 | 17.70 | 6.30 | Female | 0.08 |
| 243 | Forest | 9.70 | 5.50 | 6.30 | 4.70 | 6.20 | 4.70 | 4.10 | 1.50 | 10.63 | 11.13 | 19.90 | 8.50 | Female | 0.11 |
| 244 | Forest | 8.10 | 4.00 | 5.50 | 4.30 | 5.20 | 5.00 | 3.20 | 1.40 | 8.10 | 8.60 | 17.10 | 6.40 | Female | 0.09 |
| 245 | Forest | 7.50 | 3.70 | 8.40 | 4.00 | 5.60 | 5.10 | 3.40 | 1.30 | 8.10 | 8.80 | 16.30 | 6.00 | Male | 0.08 |
| 246 | Forest | 7.30 | 4.00 | 5.50 | 3.60 | 4.30 | 4.40 | 3.00 | 1.30 | 7.30 | 7.90 | 15.70 | 6.00 | Female | 0.10 |
| 247 | Forest | 7.50 | 3.80 | 5.30 | 3.50 | 5.00 | 4.40 | 3.10 | 1.00 | 7.60 | 8.10 | 15.70 | 6.10 | Male | 0.10 |
| 248 | Forest | 8.20 | 4.00 | 5.80 | 4.20 | 4.70 | 5.20 | 3.30 | 1.20 | 8.50 | 9.10 | 17.40 | 6.50 | Female | 0.09 |
| 249 | Forest | 7.70 | 3.40 | 5.60 | 3.40 | 4.10 | 4.70 | 3.10 | 1.50 | 7.80 | 9.00 | 15.80 | 6.90 | Female | 0.11 |
| 250 | Forest | 7.10 | 3.50 | 5.40 | 3.30 | 5.00 | 5.00 | 3.20 | 1.00 | 7.40 | 8.60 | 15.60 | 6.10 | Female | 0.09 |
| 251 | Forest | 7.50 | 3.80 | 5.60 | 3.30 | 4.50 | 4.50 | 3.20 | 0.90 | 7.90 | 8.70 | 15.80 | 6.10 | Male | 0.09 |
| 252 | Forest | 7.90 | 3.50 | 5.60 | 4.10 | 5.70 | 5.10 | 3.40 | 1.50 | 8.70 | 9.40 | 16.50 | 7.10 | Female | 0.11 |
| 253 | Forest | 8.20 | 3.40 | 5.50 | 4.10 | 4.20 | 4.50 | 3.40 | 1.40 | 7.90 | 8.70 | 16.10 | 6.00 | Female | 0.09 |
| 254 | Forest | 8.00 | 4.50 | 6.20 | 4.30 | 4.70 | 5.40 | 4.10 | 1.60 | 9.60 | 10.25 | 17.90 | 6.20 | Male | 0.07 |
| 255 | Forest | 8.30 | 4.40 | 5.10 | 3.50 | 4.90 | 5.10 | 3.10 | 1.40 | 9.10 | 9.60 | 17.80 | 7.50 | Female | 0.11 |
| 256 | Forest | 8.40 | 3.60 | 5.90 | 4.20 | 5.00 | 5.00 | 3.80 | 1.40 | 8.90 | 9.40 | 17.00 | 7.00 | Male | 0.10 |
| 257 | Forest | 8.30 | 3.60 | 5.90 | 4.30 | 4.50 | 5.10 | 3.60 | 1.50 | 8.40 | 9.20 | 17.00 | 6.80 | Female | 0.10 |
| 258 | Forest | 8.50 | 4.00 | 6.10 | 4.00 | 4.80 | 5.00 | 3.70 | 1.50 | 8.80 | 9.60 | 17.50 | 7.50 | Female | 0.11 |
| 259 | Forest | 8.10 | 3.60 | 5.60 | 4.30 | 4.50 | 4.90 | 3.20 | 1.40 | 7.90 | 8.80 | 16.60 | 6.70 | Female | 0.10 |
| 260 | Forest | 8.00 | 3.60 | 5.70 | 4.50 | 4.50 | 5.10 | 3.20 | 1.50 | 8.60 | 10.38 | 16.70 | 6.10 | Male | 0.07 |
| 261 | Forest | 8.50 | 4.80 | 6.50 | 4.40 | 5.10 | 5.90 | 4.00 | 1.60 | 10.30 | 10.63 | 19.20 | 7.50 | Male | 0.09 |
| 262 | Forest | 7.10 | 3.80 | 5.40 | 4.10 | 3.40 | 4.60 | 3.20 | 1.20 | 8.10 | 10.38 | 15.50 | 6.20 | Female | 0.08 |
| 263 | Forest | 8.70 | 3.80 | 5.70 | 4.20 | 4.50 | 5.10 | 3.30 | 1.30 | 8.50 | 9.20 | 17.60 | 6.60 | Female | 0.09 |
| 264 | Forest | 7.70 | 4.20 | 5.70 | 4.30 | 4.30 | 5.00 | 3.70 | 1.30 | 8.50 | 9.50 | 16.90 | 7.00 | Female | 0.10 |
| 265 | Forest | 7.20 | 3.70 | 5.90 | 4.20 | 4.10 | 5.00 | 3.90 | 1.50 | 8.90 | 9.50 | 15.90 | 6.70 | Male | 0.10 |
| 266 | Forest | 8.00 | 4.30 | 6.00 | 4.10 | 5.20 | 5.60 | 4.40 | 1.50 | 9.70 | 10.50 | 17.90 | 6.30 | Male | 0.07 |
| 267 | Forest | 8.70 | 4.10 | 5.70 | 4.10 | 4.20 | 5.20 | 3.50 | 1.40 | 8.10 | 8.90 | 18.00 | 6.80 | Female | 0.10 |
| 268 | Forest | 8.50 | 3.80 | 5.50 | 4.50 | 3.70 | 4.90 | 3.60 | 1.40 | 8.50 | 9.20 | 17.20 | 7.00 | Female | 0.10 |
| 269 | Forest | 8.00 | 3.20 | 6.40 | 4.10 | 6.00 | 5.60 | 4.20 | 1.40 | 9.70 | 9.90 | 16.80 | 5.80 | Male | 0.07 |
